# Aluminum Salen Complexes Modified with Unsaturated Alcohol: Synthesis, Characterization, and Their Activity towards Ring-Opening Polymerization of ε-Caprolactone and *D*,*L*-Lactide

**DOI:** 10.3390/molecules28031262

**Published:** 2023-01-27

**Authors:** Kirill V. Zaitsev, Andrey D. Trubachev, Yuri F. Oprunenko, Yuliya A. Piskun, Irina V. Vasilenko, Andrei V. Churakov, Sergei V. Kostjuk

**Affiliations:** 1 Department of Chemistry, Moscow State University, Leninskye Gory 1, 3, Moscow 119991, Russia; 2 Research Institute for Physical Chemical Problems of the Belarusian State University, Leningradskaya Str., 14, 220006 Minsk, Belarus; 3 Faculty of Chemistry, Belarusian State University, Leningradskaya Str., 14, 220006 Minsk, Belarus; 4 N.S. Kurnakov Institute of General and Inorganic Chemistry, Russian Academy of Sciences, Leninskii Pr., 31, Moscow 119991, Russia; 5 Institute for Regenerative Medicine, Sechenov First Moscow State Medical University, 8-2, Trubetskaya Str., Moscow 119992, Russia

**Keywords:** salen, imine ligands, aluminum complex, aluminum alkoxide, NMR spectroscopy, X-ray diffraction analysis, ring-opening polymerization, macromonomer

## Abstract

A highly efficient one-step approach to the macromonomer synthesis using modified aluminum complexes as catalysts of ring-opening polymerization (ROP) of ε-caprolactone and *D*,*L*-lactide was developed. The syntheses, structures, and catalytic activities of a wide range of aluminum salen complexes, **3a-c**, functionalized with unsaturated alcohol (HO(CH_2_)_4_OCH=CH_2_) are reported. X-Ray diffraction studies revealed a tetragonal pyramidal structure for **3c**. Among the complexes **3a**-**c**, the highest activity in bulk ROP of ε-caprolactone and *D*,*L*-lactide was displayed by **3b**, affording polyesters with controlled molecular weights at low monomer to initiator ratios (*M_n_* up to 15,000 g mol^−1^), relatively high polydispersities (Ð~1.8) and high number-average functionalities (*F_n_* up to 85%).

## 1. Introduction

Aluminum alkoxides, due to their low toxicity, high Lewis acidity, wide abundance, and low cost, find application in chemistry, material science, and related fields of science. Examples are numerous catalysts in organic synthesis [1,2,3,4,5,6,7], the activation of small molecules (such as carbon dioxide) [8,9,10,11], description of the synthesis of complexes with unusual structures [12,13], precursors for sol-gel technology [14] or OLED and optic materials [15,16,17] based on Al compounds. At the same time, there are many works concerning the application of aluminum alkoxides based on different ligands as initiators [18,19,20,21,22,23] in ring-opening polymerization (ROP) [24,25,26,27,28] of lactones and lactide for the synthesis of biodegradable polymers. This method of polymerization differs from an alternative way of preparing polyesters such as polycondensation [29,30] by a more controlled character of polymerization that allows obtaining (co)polymers with target molar mass and low polydispersity as well as high end-group fidelity. Beginning with works by Spassky [31], Al complexes based on salen (bis(salicylaldiimine)) type ligands [20,32,33,34,35,36,37,38,39,40,41,42,43,44,45,46,47,48,49,50,51,52,53,54] found wide application in this field due to simple synthesis, various structures, and good polymerization control. Moreover, Atwood et al. [55] continue to apply Al salen complexes in organometallic and coordination chemistry. Despite a significant progress in this field of science, the development of new catalysts with precise structure, which may be used for construction of new materials, is a challenge for modern chemistry.

Furthermore, the construction of complex polymer architectures requires new methodologies for material synthesis. Elaboration of novel macromonomers may be regarded as one of such ways [56]. In this case, the macromonomer is a polymeric molecule suitable for subsequent polymerization by a different polymerization mechanism. Therefore, a macromonomer synthesized by ROP of lactones or lactide may be used in polymerization by a radical mechanism to yield graft-copolymers [57]. In general, this concept of catalysis enables the formation of polymer materials of different architectures (branched, star-shape, brushes, dendrimers, etc.) [57,58]. Furthermore, the working out of well-established ways for synthesis of macromonomers based on polyesters may be regarded as a significant challenge, especially in comparison with similar compounds based on polyolefins [59]. Due to the fact that the specific structures, properties and aggregation behavior due to the presence of hydrophobic and hydrophilic parts, these polymer architectures are suitable for a range of applications, for example, in such fields as drug delivery systems, coatings, optic materials, etc. [60,61,62,63].

**Scheme 1:**
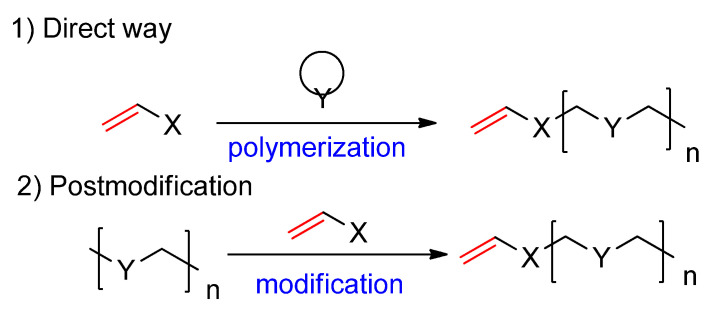
Ways of macromonomer synthesis.

To date, there are generally two main approaches to macromonomer synthesis (Scheme 1). The first includes the direct growth using a functionalized initiator, usually a simple organic compound (or more elaborate ways, i.e., atom transfer radical polymerization, ATRP; reversible addition−fragmentation chain transfer, RAFT; or catalytic chain transfer polymerization, CCTP) in the presence of a catalyst [64,65,66,67]. At the same time, it should be mentioned that the isolation of individual catalysts is more significant than the investigation of catalytically active metal systems based on modified reagents [68,69,70,71,72,73,74,75]. The second way is to use the post-polymerization modification of previously obtained polymers [76,77,78,79,80,81,82]. 

**Scheme 2:**
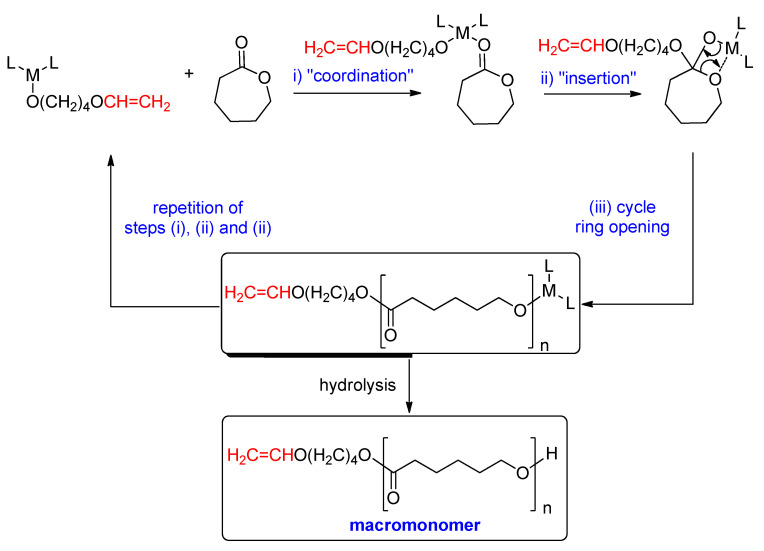
Mechanism of macromonomer synthesis.

In accordance with the single-site catalyst theory, the “insertion—coordination” mechanism (compare with the “activated monomer mechanism”) [83] has been accepted for the ROP of lactones. In continuation of our research [84,85] in this field, we propose the directly controlled synthesis of macromonomers (Scheme 2). It should be noted that this synthesis strategy proposes the application of isolated and characterized metal initiators. This technique differs significantly from other ways of end-chain-modified macromonomer synthesis.

According to this mechanism, the alkoxide group initially present at the Al atom is transferred to the end of the polymer chain. Using this approach, we may obtain end-functionalized macromonomers if the initiator, L_2_M(O(CH_2_)_4_OCH=CH_2_) (where L is a polydentate ligand), possesses the fragment suitable for subsequent polymerization. The framework of vinyl ether may be regarded as such a fragment [85]. Furthermore, the application of vinyl ether-terminated macromonomers is suitable for further polymerization [86,87,88].

This research combines investigations in several related fields of chemistry including organometallic synthesis and polymer chemistry. We describe the synthesis of new Al salen complexes (**3a-c**) containing a fragment of unsaturated alcohol. These complexes were studied as initiators of ROP of lactones and lactide under different conditions resulting in macromonomers with controlled molar masses under solvent-free (bulk) polymerization conditions. Thus, the aim of this research is the application of available and cheap salen ligands for the synthesis of Al complexes and their use as initiators for macromonomer synthesis.

## 2. Results

### 2.1. Synthesis of Ligands and Complexes

**Scheme 3:**
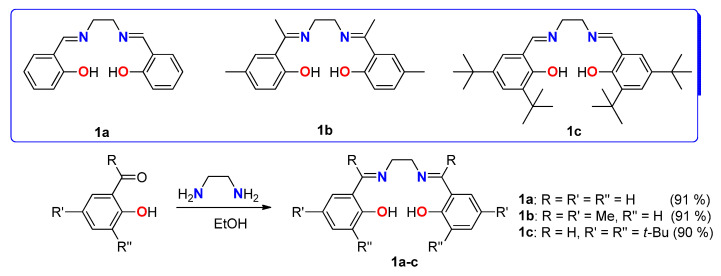
The structures of ligands **1a-c**.

The salen ligands **1a–c** (Scheme 3) differ by the presence of alkyl substituents in phenolic and iminic parts. The simplicity of their synthesis [89] (the reaction of ethylenediamine with the corresponding carbonyl compound in a 1:2 molar ratio) and high yields (more than 90%) are the main advantages of applying salen compounds. Ligand **1b** is a new compound.

The strategy including two stages was chosen for the synthesis of target-modified aluminum complexes. At the first stage, the corresponding methyl complexes, SalenAlMe, **2a–c**, would be obtained. At the second stage, the methyl group would be substituted by unsaturated alcohol, giving the target **3a–c**.

**Scheme 4:**
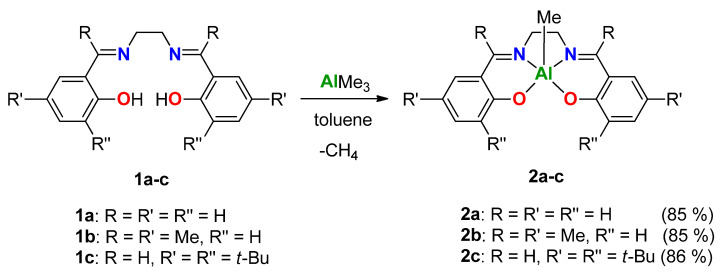
Synthesis of methyl aluminum complexes **2a-c**.

Complexes **2a-c** were obtained by the interaction of the corresponding ligands with a solution of trimethylaluminum in equimolar amounts (Scheme 4). This reaction is performed in mild conditions giving the target compounds with high yields (more than 86%). It should be noted that only a gaseous by-product, methane, is produced in the reaction of alcohols and phenols with aluminum derivatives containing Al-Me bonds.

The NMR spectra of **2a–c** are typical of alkyl aluminum salen complexes. In contrast to the ligand, protons of the NCH_2_ group in the complexes are diastereotopic, resulting in two multiplets at δ = 3.5–4.0 ppm. It should be noted that the Al-Me group is observed in the ^1^H NMR spectra (δ = −1.2–−1.0 ppm), but in the ^13^C NMR spectra the signal is observed only for **2b** (δ = −10.31 ppm) (see Supporting Information for details). Compounds **2a** and **2b** are new ones.

**Scheme 5:**
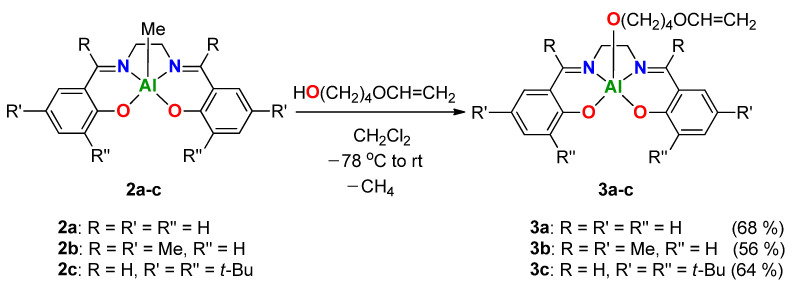
Synthesis of aluminum complexes **3a-c** containing a fragment of unsaturated alcohol.

At the second stage, the interaction of methyl complexes **2a–c** with unsaturated alcohol HO(CH_2_)_4_OCH=CH_2_ was performed in dichloromethane giving the target compounds **3a–c** in good yields (56–68%) after recrystallization (Scheme 5). These modified complexes are new compounds.

Compounds **3a-c** represent yellow powders sensitive to traces of moisture. They can be stored in a solid state under an argon atmosphere without changing. These derivatives are soluble in chloroform, dichloromethane, and THF and are sparingly soluble in toluene, and insoluble in *n-*hexane. The structure and composition of **3a-c** were established by multinuclear NMR spectroscopy, X-ray analysis, and elemental analysis data.

In solution, complexes **3a-c** have one set of signals, indicating a *C_s_*-symmetry with two equivalent iminophenolic parts. Furthermore, there is only one CRN= signal, which means that in solution these compounds are monomeric and preserve the tetragonal pyramid structure (see below XRD data for **3c**). In general, complexes **3a-c** have a common structure. Characteristic signals appear in the ^1^H/^13^C spectra at 6.30–6.40/151.9–152.0 ppm (OCH=) (for further details, see Experimental Part and Supporting Information).

### 2.2. X-ray Diffraction Analysis

**Figure 1:**
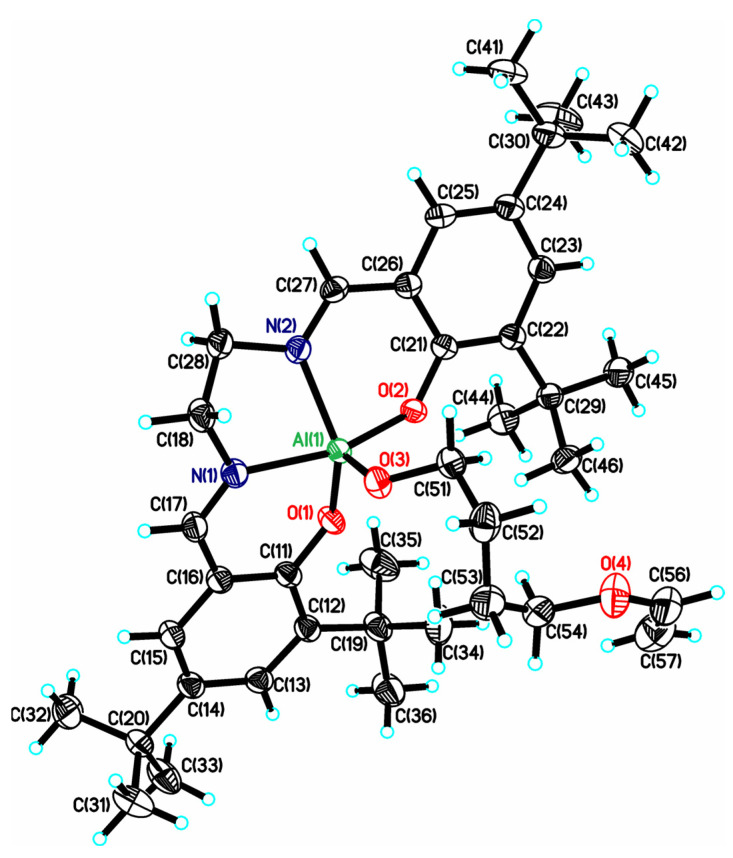
The molecular structure of complex **3c**. Displacement ellipsoids are shown at a 50% probability level. The solvated toluene molecule is omitted for clarity. Selected bond lengths (Å) and angles (deg): Al(1)-O(3) 1.7437(13), Al(1)-O(1) 1.7900(12), Al(1)-O(2) 1.8119(12), Al(1)-N(2) 2.0074(15), Al(1)-N(1) 2.0131(15) O(4)-C(56) 1.357(3), C(56)-C(57) 1.298(3); O(3)-Al(1)-O(1) 112.68(6), O(3)-Al(1)-O(2) 105.33(6), O(1)-Al(1)-O(2) 91.43(5), O(3)-Al(1)-N(2) 109.40(6), O(1)-Al(1)-N(2) 136.41(6), O(2)-Al(1)-N(2) 88.10(6), O(3)-Al(1)-N(1) 93.31(6), O(1)-Al(1)-N(1) 88.24(6), O(2)-Al(1)-N(1) 159.87(6), N(2)-Al(1)-N(1) 78.42(6), C(51)-O(3)-Al(1) 124.65(11), C(56)-O(4)-C(54) 116.44(17), C(57)-C(56)-O(4) 128.7(2).

The molecular structure of complex **3c** is given in Figure 1 (see Supporting Information, Table S1 for crystallographic information). In general, complex **3c** is not planar and has a bowl [4,8] conformation, where aryl rings are bent. Complex **3c** in a solid state is monomeric due to the presence of bulky *tert*-butyl groups (compare with dimeric methoxy substituted complexes, [(Salen)Al(μ-OMe)]_2_ [90] or [(MeSalen)Al(μ-OMe)]_2_ [91]). The aluminum atom has a geometry, which may be described as a distorted square pyramid, SP-5 (τ = 0.39; τ = 0 for an ideal square pyramid and τ = 1 for an ideal trigonal bipyramid, TBP-5) [92], where oxygen and nitrogen atoms of the salen ligand form the pyramid base. This polyhedron is typical of salen-type ligands based on ethylenediamine [93]. The oxygen atom of unsaturated alcoholate is in an axial position. The preference of tetragonal pyramidal geometry [16,21,89,93,94,95,96] in comparison with trigonal bipyramidal geometry is a common feature of *tert*-butyl substituted salen ligands. It should be noted that the Al-O bond lengths (between aluminum, salen, and unsaturated oxygen atoms) differed significantly [96,97], i.e., 1.790–1.812 vs. 1.744 Å. According to X-ray analysis, the substitution of a methyl group for unsaturated alcoholate (**2c [94]** vs. **3c**) results in a more tetragonal coordination polyhedron (the τ value decreases from 0.47 to 0.39), but the bond lengths change insignificantly. The structure of the alkoxide group also weakly affects the geometric parameters. Indeed, in the case of related [Salen(*t*-Bu)AlOEt], the Al-O_ax_ is very similar to **3c** (1.737 vs. 1.744 Å) [93,98,99]. It is interesting to note that in the case of phenoxide substituted derivatives the aluminum polyhedron is more square bipyramidal with an increased Al-O_Ar_ bond length in comparison with Al-O_Alk_ (compare with Salen(t-Bu)AlO-*p*-C_6_H_4_Ph, *d*(Al-O) 1.7609(16) Å, τ 0.61 [15]), and larger Al-O-C angle (compare 126.3–136.5° [16] in phenolate and 124.7° in alkoxy **3c**).

The catalytic activity of complexes **3a-c** in ROP of ε-caprolactone and *D*,*L*-lactide was then investigated under different conditions.

## 3. Discussion

In this section, we discuss in detail the catalytic activity of the complexes obtained as initiators in ring-opening polymerization of ε-caprolactone and *D*,*L*-lactide. 

**Figure 2:**
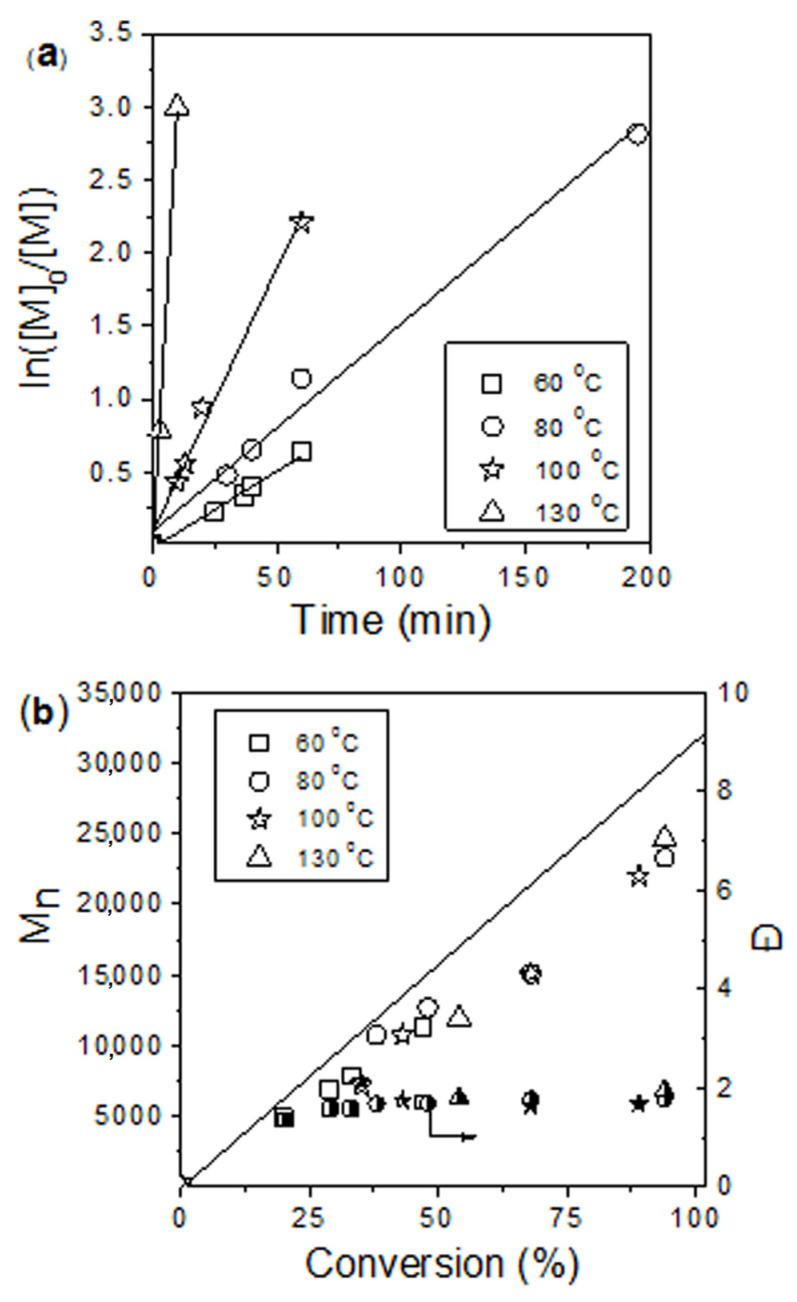
(**a**) ln[*M*]_0_/[*M*] vs. time; (**b**) *M_n_*, *Ð* vs. conversion dependences for the bulk ε-caprolactone polymerization for catalyst **3a** at [*M*]/[**3a**] = 300 and at different temperatures. The straight line in Figure 2b corresponds to the theoretical molecular weight calculated as *M_n_*(theor) = ([ε-caprolactone]/[**3a**]) × 114 × Conversion + 115. Experimental values of the molecular weight determined by SEC versus polystyrene standards were corrected by a factor of 0.52 [85].

In the initial step, the bulk ROP of ε-caprolactone was studied at different temperatures (60, 80, 100, 130 °C) using **3a** as an initiator; several elevated temperature values were used to find the optimal temperature and to clarify the polymerization kinetics. Firstly, we focused on the determination of minimal temperature, at which we can reach the complete ε-caprolactone conversion in bulk at a reasonable rate. Secondly, we were keeping in mind the possible copolymerization of ε-caprolactone with lactide, which can be performed only at 130 °C due to the high melting point of polylactide. According to Figure 2a, the polymerization rate gradually increased with increasing reaction temperature. The increase in temperature would lead to a decrease in the viscosity of the system facilitating the ε-caprolactone diffusion to the growing polymer chain-catalyst complex, on the one hand. On the other hand, the polymerization was terminated at incomplete monomer conversion at 60 °C due to the crystallization of poly(ε-caprolactone) (melting point is about 60 °C) that makes difficult or even impossible the monomer diffusion to growing polymer chain-catalyst complex. It should be noted that all first-order plots are linear, indicating the absence of irreversible termination during bulk ROP polymerization of ε-caprolactone initiated by **3a**. Construction of this plot is based on the assumption that the concentration of active species is constant (V_p_ = k_p_ × [A*] × [M], where V_p_ is the rate of propagation, k_p_—rate constant for propagation, A*- active species, [M]—monomer). Taking into account that [A*] = constant for pseudo-living/controlled polymerization, we can write V_p_ = k_p, app_ × [M], where k_p, app_—the apparent rate constant for propagation and k_p, app_ = k_p_ × [A*]). In other words, if irreversible termination will occur, the deviation from the linearity of first-order semi-logarithmic plots will be observed. Therefore, the linearity of first-order plots indicates the absence of a termination process. The number-average molecular weight of polymers obtained increased with the increase in monomer conversion, while some deviation of experimental values of *M_n_* from the theoretical line was observed at a later stage of polymerization (Figure 2b). In addition, a rise in temperature resulted in an increase in the polydispersity of poly(ε-caprolactone)s synthesized with **3a** as an initiator (Figure 2b). Based on these results, the optimal temperature for bulk ROP of ε-caprolactone was 80 °C. However, although the bulk ROP of ε-caprolactone initiated by **3a** proceeded in a controlled fashion (Figure 2), the observed deviation of experimental *M_n_* from the theoretical line (Figure 2b) in conjunction with a rather high polydispersity (Ð = 1.7–2.0) indicated that side reactions such as transesterification and/or formation of macrocycles occurred in the system. In order to suppress the side reactions, the steric bulkiness of catalyst **3a** was gradually increased by introducing methyl and *tert*-butyl substituents in *para*- (Me, **3b**) or *ortho*- and *para*- (*t*-Bu, **3c**) positions, respectively, of the benzene ring. Furthermore, for **3b** the effect of Me substituent at the iminic carbon, i.e., MeC=N, was studied.

**Figure 3:**
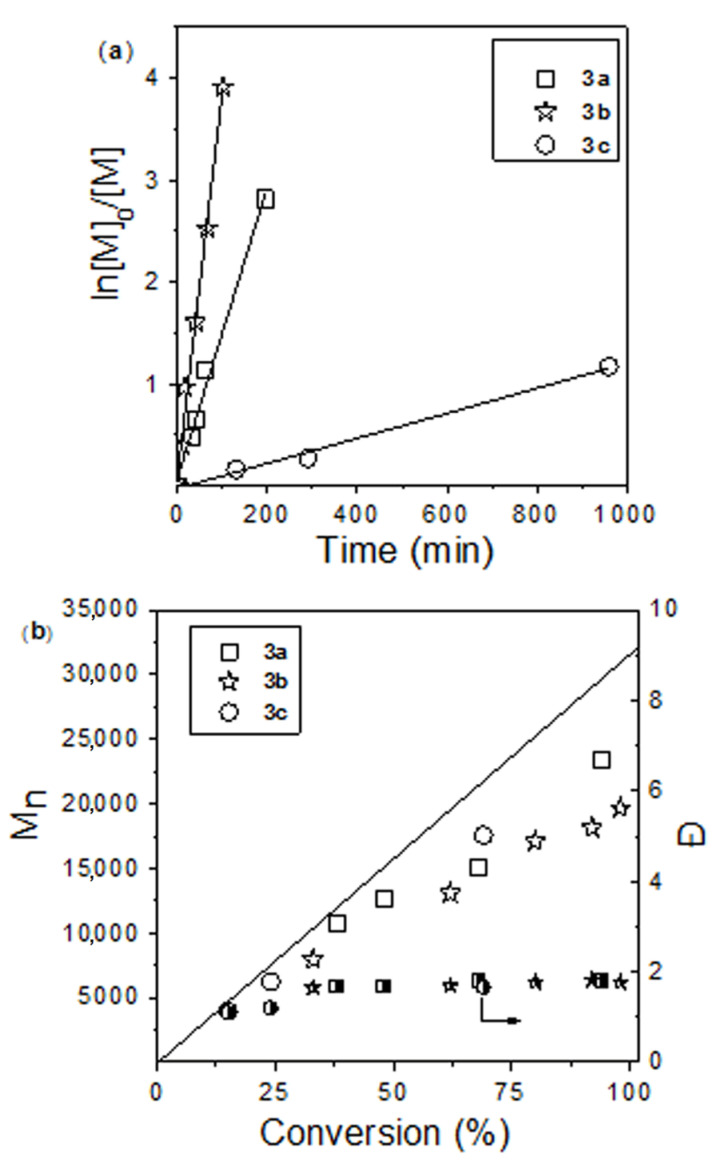
(**a**) ln[*M*]_0_/[*M*] vs. time; (**b**) *M_n_*, *Ð* vs. conversion dependences for the bulk ε-caprolactone polymerization in the presence of different catalytic complexes at [*M*]/[catalyst] = 300 and at 80 °C. The straight line in Figure 3b corresponds to the theoretical molecular weight calculated as *M_n_*(theor) = ([ε-caprolactone]/[catalyst]) × 114 × Conversion + 115. Experimental values of the molecular weight determined by SEC versus polystyrene standards were corrected by a factor of 0.52 [85].

As it can be seen from Figure 3a, the activity of investigated catalytic complexes in bulk ROP of ε-caprolactone increased in the following sequence: **3c** (*k*_p,app_ = 1.2 × 10^−3^ min^−1^) < **3a** (*k*_p,app_ = 1.4 × 10^−2^ min^−1^) < **3b** (*k*_p,app_ = 3.8 × 10^−2^ min^−1^). The observed increase in the rate of ROP of ε-caprolactone using **3b** instead of **3a** as a catalyst is surprising since the methyl substituents in the *ortho*-position of the phenyl ring and to imine nitrogen would lead to a decrease in the partial positive charge on the Al atom, which, in turn, should lead to a decrease in the reaction rate. Indeed, for related Al complexes bearing phenoxyimine ligands, the addition of electron-withdrawing substituents into aromatic ring/imine nitrogen results in an increase in the rate of ROP of ε-caprolactone in organic solvents [100,101]. However, for bulk ROP polymerization of ε-caprolactone initiated by iminophenolate aluminum complexes we observed an opposite tendency consisting of a decrease in the reaction rate upon the addition of electron-withdrawing substituents [85]. This inconsistency could be explained by the strong solvation of more electronically deficient Al atoms by the monomer under bulk polymerization conditions that may lead to the observed decrease in the reaction rate for a potentially more active catalytic complex. A much lower activity of complex **3c** in ROP of ε-caprolactone (Figure 3a) is consistent with the low accessibility of the Al atom to monomer due to the presence of bulky *tert*-butyl groups (Scheme 5).

It should be noted that the most active catalyst (**3b**) results in less controlled polymerization displaying a significant deviation of experimental values of *M_n_* from the theoretical line (Figure 3b). Moreover, the steric structure of the catalytic complex almost does not influence the polydispersity of synthesized polyesters: *Ð* lies in the range between 1.65 and 1.77 for all catalytic complexes studied (Figure 3b). This indicates that side reactions such as transesterification and/or macrocycles formation still occur in the system. Therefore, in order to confirm the controlled nature of ROP of ε-caprolactone initiated by complexes **3b** and **3c**, the polymerization was briefly investigated at different ε-caprolactone/catalyst ratios (Table 1). As it was mentioned above (*vide supra*), the *M_n_* lower than the theoretical values were obtained for all catalysts studied at [ε-caprolactone]/[catalyst] = 300 due to a side reaction such as transesterification and/or macrocycles formation. In contrast, at a lower monomer-to-catalyst ratio ([ε-caprolactone]/[catalyst] = 100), we observed a good correlation between experimental and theoretical values of *M_n_*, while the polydispersity was still relatively high (Table 1). 

**Figure 4:**
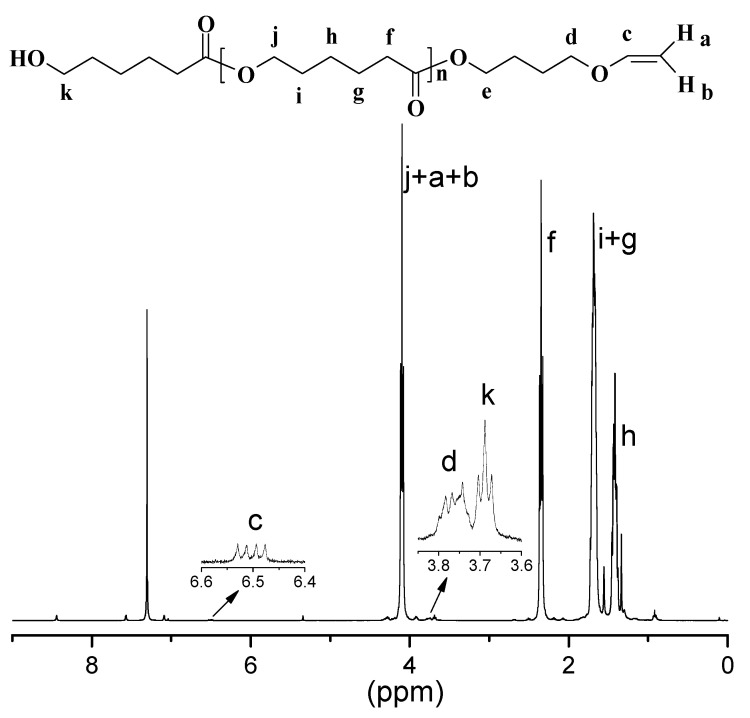
^1^H NMR spectrum of poly(ε-caprolactone) synthesized with **3b** at 80 °C and at [ε-caprolactone]/[catalyst] = 100.

It should be noted that all synthesized poly(ε-caprolactone)s are characterized by reduced functionality on the vinyl ether end group calculated from the ^1^H NMR spectrum as the integral ratio of the signal of an olefinic proton (**c**) to methylene protons (**k**) of the hydroxymethyl end group (see Figure 4). The highest functionality of 79−86% was obtained for the bulk ROP of ε-caprolactone with catalytic complexes **3b** and **3c** at a lower monomer to initiator ratio (Table 1). Evidently, transesterification, which becomes significant at a high monomer-to-initiator ratio (*vide supra*), leads to diminishing functionality at the chain end. In addition, the cationic oligomerization of obtained macromonomers during the polymerization due to the relatively high acidity of catalysts used or owing to the generation of some traces of acid during the workup procedure, could also occur taking into account the high reactivity of vinyl ethers in cationic polymerization [102]. Indeed, the functionality on the vinyl ether end group calculated as the integral ratio of protons of −CH_2_O group (**d**) to methylene protons (**k**) of the hydroxymethyl end group is higher than the one calculated based on olefinic protons. This confirms that some consumption of olefinic double bonds did take place. To finally validate this hypothesis, the aim of our further work is to investigate similar aluminum salen complexes bearing other alkenylic substituents (such as the phenoxystyrene functional group), which should yield macromonomers with higher functionality.

**Figure 5:**
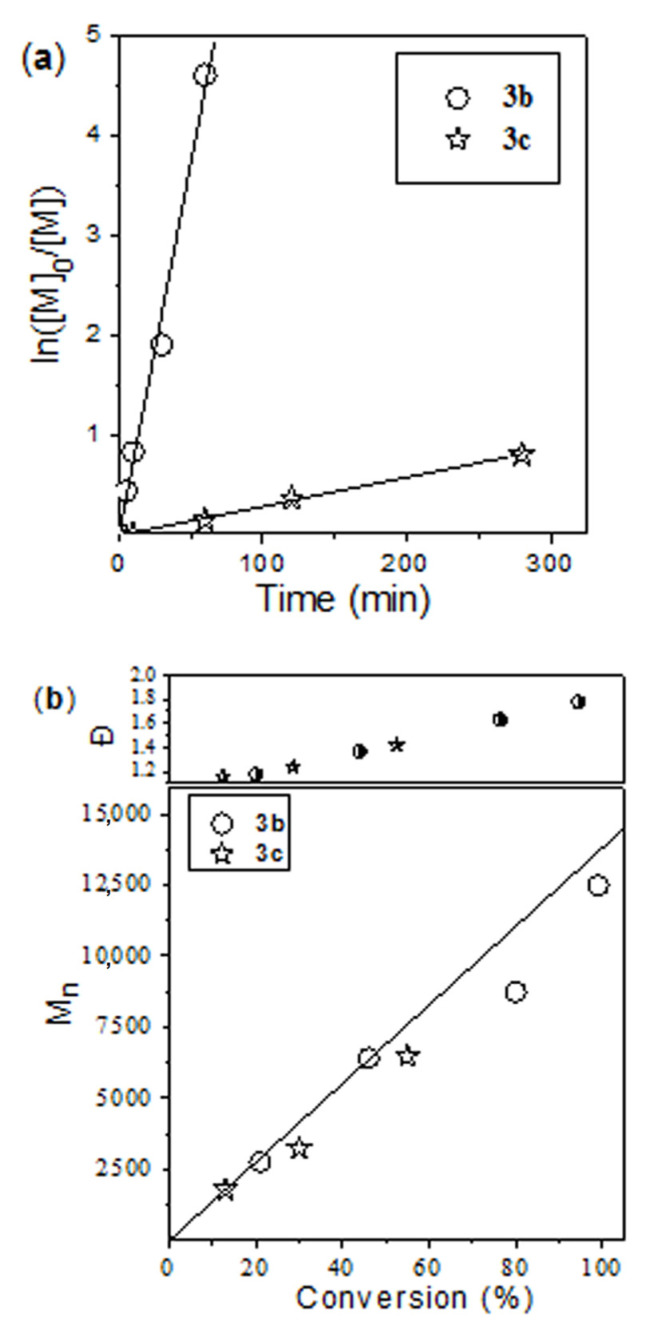
(**a**) ln[*M*]_0_/[*M*] vs. time; (**b**) *M_n_*, *Ð* vs. conversion dependences for the bulk *D*,*L*-lactide polymerization at 130 °C and [*M*]/[catalyst] = 100 for different catalytic complexes. The straight line in Figure 5b corresponds to the theoretical molecular weight calculated as *M_n_*(theor)= ([*D*,*L*-lactide]/[catalyst]) × 144 × Conversion + 115. Experimental values of the molecular weight determined by SEC versus polystyrene standards were corrected by a factor of 0.58 [85].

Taking into account the higher activity of catalyst **3b** in ROP of ε-caprolactone and better chain-end functionality for poly(ε-caprolactone)s obtained with **3b** and **3c** as initiators (*vide supra*), these two catalytic complexes were further tested in bulk ROP of *D*,*L*-lactide. Similar to the polymerization of ε-caprolactone, **3b** induced a much faster polymerization than **3c** due to the presence of bulky *tert*-butyl groups in the latter complex (compare Figure 3a and Figure 5a). On the other hand, first-order plots are linear and pass through zero (Figure 5a) indicating the absence of irreversible termination. In addition, the number-average molecular weight increases in direct proportion to monomer conversion, while the experimental values of *M_n_* correlate well with the theoretical line for both catalytic complexes studied (Figure 5b). The polydispersity of synthesized poly(*D*,*L*-lactide)s is relatively low at the beginning of the polymerization and increases up to 1.8 at the end of the reaction, while for poly(ε-caprolactone) obtained using the same catalytic complexes the polydispersity is rather high even at the beginning of polymerization (compare Figure 2b and Figure 5b). This indicates that the polymerization of *D*,*L*-lactide is more controlled than that of ε-caprolactone under similar conditions, and side reactions such as transesterification and macrocyclization are significantly suppressed in the case of the ROP of *D*,*L*-lactide.

**Figure 6:**
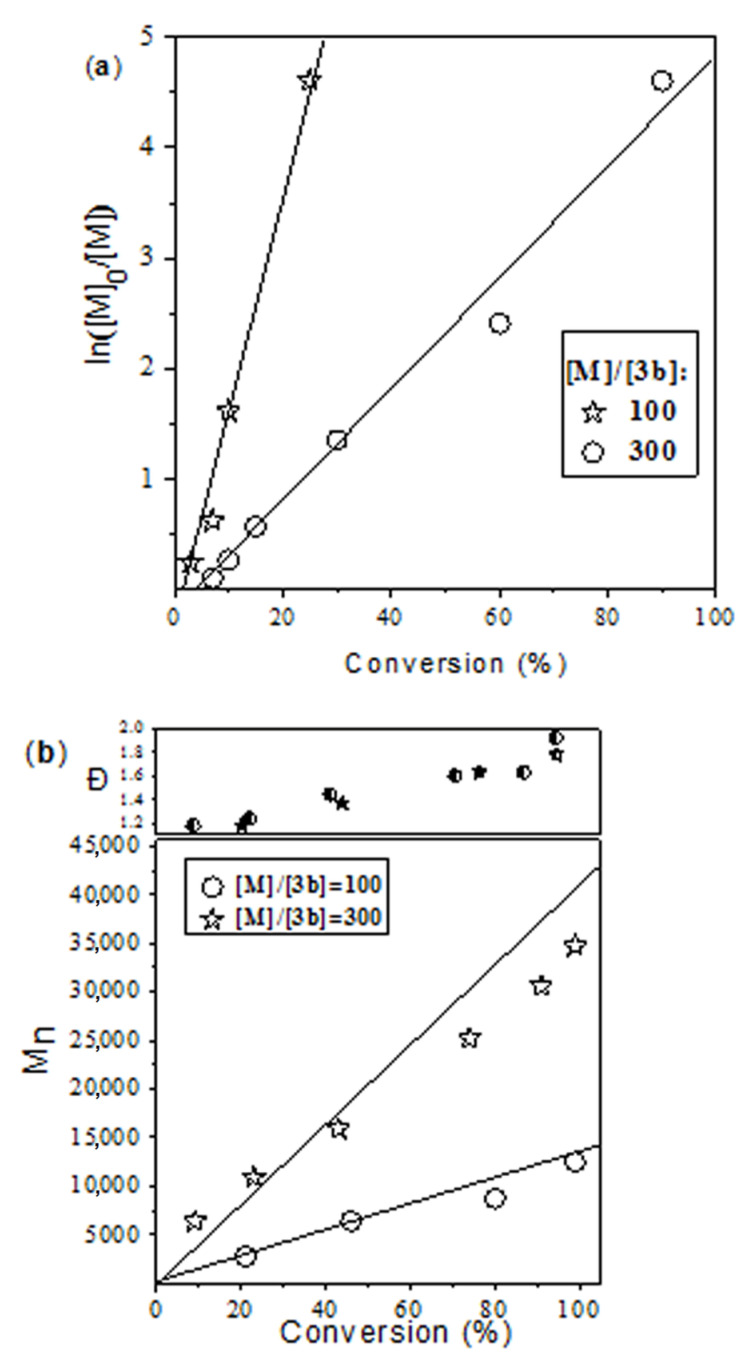
(**a**) ln[*M*]_0_/[*M*] vs. time; (**b**) *M_n_*, *Ð* vs. conversion dependences for the bulk *D*,*L*-lactide polymerization for complex **3b** at 130 °C and at different [*M*]/[catalyst] ratios. The straight lines in Figure 6b correspond to the theoretical molecular weight calculated as *M_n_*(theor) = ([*D*,*L*-lactide]/[catalyst]) × 144 × Conversion + 115. Experimental values of the molecular weight determined by SEC vs. polystyrene standards were corrected by a factor of 0.58 [85].

Since no significant difference in polydispersity for poly(*D*,*L*-lactide)s synthesized with **3b** and **3c** as catalysts was observed, the more active complex **3b** was used in further investigations. To confirm the pseudo-living nature of bulk ROP of *D*,*L*-lactide initiated by **3b**, the polymerization was investigated at two different monomer-to-catalyst ratios. As it is shown in Figure 6, the ROP of *D*,*L*-lactide initiated by **3b** at different monomer-to-catalyst ratios proceeds in a pseudo-living fashion: first-order plots are linear, while the number-average molecular weight increases in direct proportion to monomer conversion. In addition, the experimental values of *M_n_* are in good agreement with the theoretical line, while polydispersity becomes high only at the later steps of polymerization.

It should be noted that for poly(*D*,*L*-lactide)s synthesized with **3b** and **3c**, similar to poly(ε-caprolactone) prepared using the same catalysts, we observed a reduced functionality on the vinyl ether end group calculated from ^1^H NMR spectra (*F_n_* = 75% − 85%, see Table S2, Figure S19). This reduced functionality, as we showed above, was consistent with the high activity of the vinyl ether end group in cationic polymerization. Therefore, highly electron-deficient aluminum atoms in **3a**−**c** or tracers of acid generated during the workup procedure may initiate the cationic oligomerization of prepared macromonomers leading to the observed decrease in functionality. 

## 4. Materials and Methods

**General Methods and Instrumentation.** All manipulations with aluminum compounds were carried out using standard Schlenk techniques under an argon atmosphere. Solvents were purified using the usual procedures. Diethyl ether and THF were stored under solid KOH and then distilled under sodium/benzophenone. Dichloromethane and ethanol were refluxed and distilled over CaH_2_. Benzene, and *n-*hexane were refluxed over sodium and distilled off. Toluene (reagent grade) was treated with H_2_SO_4_, washed with aqueous NaHCO_3_ and distilled water to a neutral reaction, dried with CaCl_2_, refluxed, and distilled with metallic sodium. Then, it was refluxed with Na/benzophenone to a blue color and distilled under an argon atmosphere. Deuterated solvent (CDCl_3_) was dried over CaH_2_, distilled and stored under argon. The ^1^H NMR (400 MHz) and ^13^C NMR (100 MHz) spectra were recorded with a Bruker 400 spectrometer (at 295 K). Chemical shifts are given in ppm relative to internal Me_4_Si. Elemental analyses were carried out by the Microanalytical Laboratory of the Chemistry Department, Moscow State University. Size exclusion chromatography (SEC) was performed on an Agilent 1200 apparatus with Nucleogel GPC LM-5, 300/7.7 column, and one precolumn (PL gel 5 μm guard) thermostatted at 30 °C. The detection was achieved by a differential refractometer. THF was eluted at a flow rate of 1.0 mL min^−1^. The calculation of molar mass and polydispersity was based on polystyrene standards (“Polymer Laboratories GmbH”, Darmstadt, Germany). All compounds synthesized were fully characterized by NMR spectroscopy data; their identity was proven by elemental analysis data.

**Starting Materials.** Ethylenediamine (“Aldrich”), solution of AlMe_3_ (2.0 M in toluene, “Aldrich”), 2,4-di-*tert*-butylphenol (“Aldrich”) were used as received. Salicylaldehyde (“Aldrich”), HO(CH_2_)_4_OCH=CH_2_ (“Aldrich”) were distilled before use. 3,5-Di-*tert*-butylsalicylaldehyde [103] and 2-hydroxy-5-methylacetophenone [104] were obtained using the known procedures. ε-Caprolactone (97%, “Aldrich”) was dried over CaH_2_, distilled from CaH_2_ under reduced pressure and stored under argon. *D*,*L*-Lactide (98%, “Aldrich”) was twice recrystallized from toluene and dried in a vacuum at 45 °C for 5 h. 

**Synthesis. Synthesis of the ligands.** All ligands were obtained using the standard procedure. Ethylenediamine (40.00 mmol, 1.0 eq.) was added dropwise to a solution of the corresponding carbonyl compound (80.00 mmol, 2.0 eq.) in dry EtOH (60 mL). The reaction mixture was stirred for 8 h, and the solid formed was filtered off, washed with ethanol (2 × 10 mL), and dried in a vacuum.

**Ligand 1a, SalenH_2_.** Yellow powder, recrystallized from ethanol, m.p. 127–128 °C, m.p. 126 °C [105]. Yield 91%. ^1^H NMR (400 MHz, CDCl_3_, 25 °C): δ = 13.22 (s, 2H, OH), 8.33 (s, 2H, NCH=), 7.32–7.25 (m, 2H, aromatic protons), 7.21 (dd, *J*_H-H_ = 7.6 Hz, *J*_H-H_ = 1.5 Hz, 2H, aromatic protons), 6.94 (d, *J*_H-H_ = 8.3 Hz, 2H, aromatic protons), 6.89–6.82 (m, 2H, aromatic protons), 3.89 (s, 4H, 2NCH_2_) ppm. ^13^C NMR (100 MHz, CDCl_3_, 25 °C): δ = 166.37 (NCH=), 160.90, 132.27, 131.39, 118.57, 118.53, 116.82 (aromatic carbons), 59.59 (NCH_2_) ppm. 

**Ligand 1b, MeSalen(Me)H_2_.** Yellow powder, recrystallized from toluene/*n*-heptane mixture, m.p. 204–205 °C. Yield 91%. ^1^H NMR (400 MHz, CDCl_3_, 25 °C): δ = 15.54 (br s, 2H, OH), 7.29 (d, *J*_H-H_ = 1.5 Hz, 2H, aromatic protons), 7.09 (d, *J*_H-H_ = 1.8 Hz, 2H, aromatic protons), 7.06 (d, *J*_H-H_ = 1.8 Hz, 2H, aromatic protons), 3.94 (s, 4H, 2NCH_2_), 2.34 (s, 6H, Me), 2.27 (, 6H, Me) ppm. ^13^C NMR (100 MHz, CDCl_3_, 25 °C): δ = 172.48 (NC=), 160.71, 133.22, 128.15, 126.17, 119.05, 118.05 (aromatic carbons), 50.22 (NCH_2_), 20.63 (MeC_6_H_4_), 14.65 (MeC=) ppm. C_20_H_24_N_2_O_2_ (324.4168): calcd. C 74.04, H 7.46, N 8.64; found C 74.21, H 7.56, N 8.67.

**Ligand 1c, Salen(*t*-Bu)H_2_.** Yellow powder, m.p. 187–188 °C, m.p. 186–187 °C [106]. Yield 90%. ^1^H NMR (400 MHz, CDCl_3_, 25 °C): δ = 13.66 (s, 2H, OH), 8.39 (s, 2H, NCH=), 7.37 (d, 2H, *J*_H-H_ = 2.3 Hz, aromatic protons), 7.07 (d, 2H, *J*_H-H_ = 2.3 Hz, aromatic protons), 3.92 (s, 4H, 2NCH_2_), 1.44 (s, 18H, *t*-Bu), 1.29 (s, 18H, *t*-Bu) ppm. ^13^C NMR (100 MHz, CDCl_3_, 25 °C): δ = 167.57 (NCH=), 158.01, 140.03, 136.57, 126.99, 126.04, 117.79 (aromatic carbons), 59.59 (NCH_2_), 35.00 (CMe_3_), 34.08 (CMe_3_), 31.46 (CMe_3_), 29.41 (CMe_3_) ppm.

**Synthesis of the aluminum salen complexes containing the methyl group at the Al atom.** All complexes were obtained using the standard procedure. At −78 °C, a solution of AlMe_3_ in toluene (2.10 mL, 2.0 M, 7.00 mmol) was added dropwise to the solution of the corresponding ligand (7.00 mmol, 1.0 eq.) in CH_2_Cl_2_ (30 mL). The reaction mixture was slowly warmed to room temperature, then stirred overnight and refluxed for 2 h. All volatile materials were removed under reduced pressure; the residue was treated with *n-*hexane and a small amount of ether, and cooled to −30 °C. The powder formed was isolated by filtration and dried in a vacuum. 

**Complex 2a, (Salen)AlMe.** Yellowish powder. Yield 85%. ^1^H NMR (400 MHz, CDCl_3_, 25 °C): δ = 8.20 (s, 2H, NCH=), 7.39–7.33 (m, 2H, aromatic protons), 7.13 (dd, *J*_H-H_ = 7.8 Hz, *J*_H-H_ = 1.4 Hz, 2H, aromatic protons), 7.06 (d, *J*_H-H_ = 8.3 Hz, 2H, aromatic protons), 6.73–6.65 (m, 2H, aromatic protons), 3.98–3.91 (m, 2H, NCH_2_), 3.68–3.61 (m, 2H, NCH_2_) ppm. ^13^C NMR (100 MHz, CDCl_3_, 25 °C): δ = 168.50 (NCH=), 165.82, 135.58, 133.03, 122.69, 118.76, 116.41 (aromatic carbons), 54.58 (NCH_2_) ppm. In the ^13^C NMR spectrum the signal of the AlMe group was not found. C_17_H_17_AlN_2_O_2_ (308.3106): calcd. C 66.23, H 5.56, N 9.09; found C 65.53, H 5.36, N 8.87.

**Complex 2b, (MeSalen(Me))AlMe.** Yellowish powder. Yield 85%. ^1^H NMR (400 MHz, CDCl_3_, 25 °C): δ = 7.24 (s, 2H, aromatic protons), 7.11, 6.95 (2d, *J*_H-H_ = 8.6 Hz, each 2H, aromatic protons), 3.82–3.70 (m, 4H, 2NCH_2_), 2.42 (s, 6H, Me), 2.22 (s, 6H, Me), −1.18 (s, 3H, AlMe) ppm. ^13^C NMR (100 MHz, CDCl_3_, 25 °C): δ = 174.85 (NC=), 162.93, 135.47, 128.81, 124.37, 123.45, 120.02 (aromatic carbons), 48.33 (NCH_2_), 20.54 (MeC_6_H_4_), 18.28 (MeC=), −10.31 (AlMe) ppm. C_21_H_25_AlN_2_O_2_ (364.4169): calcd. C 69.21, H 6.91, N 7.69; found C 68.78, H 6.77, N 7.73.

**Complex 2c, (Salen(*t*-Bu))AlMe.** Yellow powder. Yield 86%. ^1^H NMR (400 MHz, CDCl_3_, 25 °C): δ = 8.29 (s, 2H, NCH=), 7.51 (d, 2H, *J*_H-H_ = 2.3 Hz, aromatic protons), 7.00 (d, 2H, *J*_H-H_ = 2.3 Hz, aromatic protons), 3.98–3.91 (m, 2H, 2NCH_2_), 3.72–3.59 (m, 2H, 2NCH_2_), 1.54 (s, 18H, *t*-Bu), 1.30 (2s, each 18H, *t*-Bu), −1.10 (s, 3H, AlMe) ppm. ^1^H NMR corresponds to the literature data [94]. ^13^C NMR (100 MHz, CDCl_3_, 25 °C): δ = 169.01 (NCH=), 163.54, 141.08, 137.48, 130.42, 126.94, 118.30 (aromatic carbons), 55.17 (NCH_2_), 35.58 (CMe_3_), 33.93 (CMe_3_), 31.39 (CMe_3_), 29.68 (CMe_3_) ppm. In the ^13^C NMR spectrum the signal of the AlMe group was not found.

**Synthesis of the aluminum salen complexes containing a fragment of unsaturated alcohol.** All complexes were obtained using the standard procedure. At −78 °C, HO(CH_2_)_4_OCH=CH_2_ (0.17 mL, 1.35 mmol) was added dropwise to the solution of the corresponding methyl aluminum complex (1.35 mmol, 1.0 eq.) in CH_2_Cl_2_ (30 mL). The reaction mixture was slowly warmed to room temperature and then stirred overnight. All volatile materials were removed under reduced pressure; the residue was treated with *n-*hexane and a small amount of ether, and cooled to −30 °C. The powder formed was isolated by filtration and dried in a vacuum. 

**Complex 3a, (Salen)Al(O(CH_2_)_4_OCH=CH_2_).** Yellow powder. Yield 68%. ^1^H NMR (400 MHz, CDCl_3_, 25 ^o^C): δ = 8.29 (s, 2H, NCH=), 7.42–7.35 (m, 2H, aromatic protons), 7.16 (d, *J*_H-H_ = 6.8 Hz, 2H, aromatic protons), 7.10 (d, *J*_H-H_ = 7.8 Hz, 2H, aromatic protons), 6.73 (t, *J*_H-H_ = 7.2 Hz, 2H, aromatic protons), 6.34 (dd, *J*_H-H_ = 14.3 Hz, *J*_H-H_ = 6.7 Hz, 1H, OCH=CH_2_), 4.10–4.03 (m, 3H, OCH_2_, OCH=CH(H)), 3.87 (br d, *J*_H-H_ = 6.7 Hz, 1H, OCH=CH(H)), 3.71 (br s, 2H, OCH_2_), 3.51–3.44 (m, 4H, 2NCH_2_), 1.48 (t, *J*_H-H_ = 6.6 Hz, 2H, CH_2_), 1.36–1.32 (m, 2H, CH_2_) ppm. ^13^C NMR (100 MHz, CDCl_3_, 25 °C): δ = 169.18 (NCH=), 151.99 (OCH=), 165.91, 135.78, 133.02, 122.50, 118.79, 116.84 (aromatic carbons), 85.97 (=CH_2_), 68.43, 62.38 (2OCH_2_), 54.70 (2NCH_2_), 30.96 (CH_2_), 25.94 (CH_2_) ppm. C_22_H_25_AlN_2_O_4_ (408.4264): calcd. C 64.70, H 6.17, N 6.86; found C 64.23, H 5.96, N 6.67.

**Complex 3b, (MeSalen(Me))Al(O(CH_2_)_4_OCH=CH_2_).** Yellow powder. Yield 56%. ^1^H NMR (400 MHz, CDCl_3_, 25 °C): δ = 7.27 (br s, 2H, NCH=), 7.14 (d, *J*_H-H_ = 8.2 Hz, 2H, aromatic protons), 6.99 (d, *J*_H-H_ = 8.2 Hz, 2H, aromatic protons), 6.35 (br s, 1H, OCH=), 4.08–4.02 (m, 1H, =CH(H)), 3.98–3.90 (m, 2H, OCH_2_), 3.88 (br s, 1H, =CH(H)), 3.82–3.76 (m, 2H, OCH_2_), 3.43 (br s, 4H, 2NCH_2_), 2.48 (s, 6H, 4Me), 2.23 (s, 6H, 4Me), 1.42 (br s, 2H, CH_2_), 1.32–1.24 (m, 2H, CH_2_) ppm. ^13^C NMR (100 MHz, CDCl_3_, 25 °C): δ = 157.97 (NC=), 151.93 (OCH=), 163.06, 135.79, 128.88, 124.77, 123.27, 119.81 (aromatic carbons), 85.92 (OCH=CH_2_), 68.41 (OCH_2_), 62.24 (OCH_2_), 48.55 (2NCH_2_), 31.51 (CH_2_), 25.55 (CH_2_), 20.59 (MeC_6_H_3_), 18.41 (=CMe) ppm. C_26_H_33_AlN_2_O_4_ (464.5328): calcd. C 67.22, H 7.16, N 6.03; found C 66.56, H 6.87, N 5.87.

**Complex 3c, (Salen(*t*-Bu)Al(O(CH_2_)_4_OCH=CH_2_).** Yellow powder. Yield 64%. ^1^H NMR (400 MHz, CDCl_3_, 25 °C): δ = 8.36 (s, 2H, NCH=), 7.50 (d, *J*_H-H_ = 2.3 Hz, 2H, aromatic protons), 7.00 (d, *J*_H-H_ = 2.3 Hz, 2H, aromatic protons), 6.31 (dd, *J*_H-H_ = 14.4 Hz, *J*_H-H_ = 6.8 Hz, 1H, OCH=), 4.12–4.05 (m, 2H, OCH_2_), 4.00 (dd, 1H, *J*_H-H_ = 14.4 Hz, *J*_H-H_ = 1.4 Hz, OCH(H)=), 3.85 (dd, 1H, *J*_H-H_ = 6.8 Hz, *J*_H-H_ = 1.4 Hz, OCH(H)=), 3.72–3.66 (m, 2H, OCH_2_), 3.46–3.37 (m, 4H, 2NCH_2_), 1.51–1.42 (m, 2H, 2CH_2_), 1.34–1.29 (m, 2H, 2CH_2_), 1.54 (s, 18H, 2CMe_3_), 1.29 (s, 18H, 2CMe_3_) ppm. ^13^C NMR (100 MHz, CDCl_3_, 25 °C): δ = 169.60 (NCH=), 151.97 (OCH=), 163.49, 140.75, 138.00, 130.60, 127.07, 118.26 (aromatic carbons), 85.89 (OCH_2_=), 68.37 (OCH_2_), 61.94 (OCH_2_), 55.06 (NCH_2_) 35.60 (CMe_3_), 33.94 (CMe_3_), 31.36 (CMe_3_), 29.75 (CMe_3_), 30.94 (CH_2_), 25.57 (CH_2_) ppm. C_38_H_57_AlN_2_O_4_ (632.8517): calcd. C 72.12, H 9.08, N 4.43; found C 71.89, H 8.93, N 4.34.

**X-Ray diffraction study.** Experimental intensities were collected on a Bruker Smart Apex II diffractometer (graphite monochromatized Mo-*K*α radiation, λ = 0.71073 Å) using *ω*-scan mode. Absorption correction based on measurements of equivalent reflections (SADABS) was applied. The structure was solved by direct methods and refined by full- matrix least-squares on *F*^2^ (Shelxtl) with anisotropic thermal parameters for all non-hydrogen atoms. The solvent toluene molecule was found to be disordered over three positions with occupancy ratios 0.477(3)/0.290(3)/0.233(3). All H atoms were placed in calculated positions and refined using a riding model with freely rotating methyl groups. Details are listed in Table S1. Crystallographic data have been deposited with the Cambridge Crystallographic Data Centre as supplementary publication no. CCDC-2220903.

**Polymerization Procedures**. Bulk ROP*s* of ε-caprolactone and *D*,*L*-lactide were carried out in Schlenk reactors equipped with magnetic stirring bars under an argon atmosphere. For kinetic studies, the reaction mixtures were regularly sampled throughout the polymerization. The samples were promptly cooled to ~20 °C to stop the polymerization. The conversion of the monomer was determined from ^1^H NMR spectra.

**Polymerization of ε-caprolactone** (**CL)**. The bulk ring-opening polymerization of ε-caprolactone was carried out as follows ([M]/[catalyst] = 300): a 10 mL reactor was charged with a 0.1 M solution of the catalyst in toluene (1.57 mL, 1.57 × 10^–4^ mol). The solvent was removed in a vacuum at ~20 °C for 5 min, and CL (5 mL, 0.047 mol) was added. The reactor was immersed in an oil bath preheated to the desired temperature to start the polymerization.

**Polymerization of *D*,*L*-Lactide (LA)**. The bulk ring-opening polymerization of *D*,*L*-lactide was carried out as follows ([*M*]/[catalyst] = 300): a 10 mL reactor was charged with 5.00 g (0.035 mol) of *D*,*L*-lactide, then a 0.1 M solution of the catalyst in toluene (1.57 mL, 1.57 ×10^–4^ mol) was added. The solvent was removed in a vacuum at ~20 °C for 5 min. The reactor was immersed in an oil bath preheated to 130 °C to start the polymerization.

## 5. Conclusions

Here, we reported the first comprehensive investigation of the synthesis, structure, and ROP catalytic activity of a number of mononuclear well-defined aluminum salen complexes, **3a-c**, containing unsaturated alcoholate as a coligand. Among complexes **3a**−**c**, the highest catalytic activity in bulk ROP of ε-caprolactone and *D*,*L*-lactide was shown by complex **3b** bearing methyl substituents in *para*-position of the phenyl ring and at the imine carbon atom. These electron donating substituents would lead to a decrease in the acidity of the complex’s Al atom that theoretically would decrease the activity of the catalyst in ROP. However, strong solvation of more electronically deficient Al atoms by the monomer under bulk polymerization conditions results in a retardation of polymerization for a potentially more active catalytic complex. The number-average functionality of polyesters on the vinyl ether end group synthesized with **3b** and **3c** as initiators is relatively high (up to 85%) and does not depend significantly on the molecular weight of macromonomers. The catalytic strategy developed in this work uses an effective, simple and robust approach to macromonomers based on biodegradable polymers. This opens huge prospects for synthesis of various polymeric materials with new properties.

## Figures and Tables

**Table 1:** Bulk ROP of ε-caprolactone at different monomer to catalyst ratios ^1^.

Catalyst	[M]/[cat]	Time (h)	Conversion (%)	M_n_ (theor) (g mol^−1^) ^2^	M_n_ (g mol^−1^) ^3^	Ð	F_n_ (%) ^4^
**3a**	300	3.2	94	32,150	23,400	1.70	38
**3b**	300	1.7	100	34,200	19,600	1.77	63
	100	0.25	100	11,500	10,500	1.71	79
**3c**	300	16	69	23,600	17,600	1.71	68
	100	3.2	100	11,500	13,100	1.65	86

^1^ Conditions: *T* = 80 °C; Conversion was determined by ^1^H NMR spectroscopy of the reaction mixture by comparing integral intensities of signals of methylene protons on polymer and monomer, respectively. ^2^ *M_n_*(theor) = ([ε-caprolactone]/[**3a**]) × 114 × Conversion + 115. ^3^ Calculated according to the following equation: *M_n_* = 0.52 × *M_n_*(SEC). ^4^ Calculated from the ^1^H NMR spectra as follows: *F_n_* = 2 *I*(*c*)/*I*(*k*) × 100, see Figure 4 for assignments.

## Data Availability

Not applicable.

## Supplementary Materials

The following supporting information can be downloaded at: https://www.mdpi.com/article/10.3390/molecules28031262/s1, X-ray analysis data (Table S1), NMR spectra of the compounds obtained (Figures S1–S18), details of polymerization (Table S2, Figure S19): SI_Molecules_Zaitsev2.
