# Supplementary material for: Aluminum Salen Complexes Modified with Unsaturated Alcohol: Synthesis, Characterization, and Their Activity towards Ring-Opening Polymerization of ε-Caprolactone and D,L-Lactide
Source: Molecules. 2023 Jan 27;28(3):1262. doi: 10.3390/molecules28031262 (PMC9920203; doi:10.3390/molecules28031262)
Supplement: Supplementary file 1 [file molecules-28-01262-s001.zip › molecules-2094886-supplementary.pdf]

Aluminum Salen Complexes Modified with Unsaturated Alcohol: Synthesis, Characterization, and Their Activity towards Ring-Opening Polymerization of ε-Caprolactone and *D,L*-Lactide

Kirill V. Zaitsev,[1,*] Andrey D. Trubachev,[1] Yuri F. Oprunenko,[1] Yuliya A. Piskun,[2] Irina V. Vasilenko,[2,3,*] Andrei V. Churakov,[4] Sergei V. Kostjuk[2,3,5,*]

[1] Department of Chemistry, Moscow State University, Leninskye Gory 1, 3, Moscow 119991, Russia

[2] Research Institute for Physical Chemical Problems of the Belarusian State University, Leningradskaya Str., 14, 220006, Minsk, Belarus

[3] Faculty of Chemistry, Belarusian State University, Leningradskaya Str., 14, 220006, Minsk, Belarus

[4] N.S. Kurnakov Institute of General and Inorganic Chemistry, Russian Academy of Sciences, Leninskii Pr., 31, Moscow 119991, Russia

[5] Institute for Regenerative Medicine, Sechenov First Moscow State Medical University, 8-2, Trubetskaya Str., 119992 Moscow, Russia

Correspondence to: K.V. Zaitsev (E-mail: *zaitsev@org.chem.msu.ru*) or S.V. Kostjuk (E-mail: *kostjuks@bsu.by*) or I.V. Vasilenko (E-mail: *vasilenkoi@bsu.by*).

Supporting Information

X-ray analysis data

Table S1. The crystallographic data for compound **3c**…..S3

NMR Spectra

Figure S1. ^{1}H NMR spectrum of **1a** ($CDCl_3$, RT)...S4
Figure S2. ^{13}C NMR spectrum of **1a** ($CDCl_3$, RT)...S4
Figure S3. ^{1}H NMR spectrum of **1b** ($CDCl_3$, RT)...S5
Figure S4. ^{13}C NMR spectrum of **1b** ($CDCl_3$, RT)...S5
Figure S5. ^{1}H NMR spectrum of **1c** ($CDCl_3$, RT)...S6

Figure S6. ^{13}C APT NMR spectrum of **1c** ($CDCl_3$, RT)……S6
Figure S7. ^{1}H NMR spectrum of **2a** ($CDCl_3$, RT)……S7
Figure S8. ^{13}C NMR spectrum of **2a** ($CDCl_3$, RT)……S7
Figure S9. ^{1}H NMR spectrum of **2b** ($CDCl_3$, RT)……S8
Figure S10. ^{13}C NMR spectrum of **2b** ($CDCl_3$, RT)……S8
Figure S11. ^{1}H NMR spectrum of **2c** ($CDCl_3$, RT)……S9
Figure S12. ^{13}C NMR spectrum of **2c** ($CDCl_3$, RT)……S9
Figure S13. ^{1}H NMR spectrum of **3a** ($CDCl_3$, RT)……S10
Figure S14. ^{13}C NMR spectrum of **3a** ($CDCl_3$, RT)……S10
Figure S15. ^{1}H NMR spectrum of **3b** ($CDCl_3$, RT)……S11
Figure S16. ^{13}C NMR spectrum of **3b** ($CDCl_3$, RT)……S11
Figure S17. ^{1}H NMR spectrum of **3c** ($CDCl_3$, RT)……S12
Figure S18. ^{13}C NMR spectrum of **3c** ($CDCl_3$, RT)……S12

Polymerization data

Table S2. Bulk polymerization of *D*,*L*-lactide at different monomer/catalyst ratios for **3b** and **3c** at 130 ºC……S13
Figure S19. ^{1}H NMR spectrum of poly(*D*,*L*-lactide) synthesized with **3b** at 130 °C and at [*D*,*L*-lactide]/[catalyst] = 100……S14

X-ray analysis data

Table S1. The crystallographic data for compound **3c**.

empirical formula	$C_{38}H_{57}AlN_2O_4 \cdot C_7H_8$
M_w	724.97
temperature (K)	173
size (mm)	0.25 x 0.20 x 0.15
cryst. system	monoclinic
space group	$P2_1/c$
a (Å)	13.2610(10)
b (Å)	26.638(2)
c (Å)	13.4757(10)
β (deg)	117.049(1)
V (Å^3)	4239.5(6)
Z	4
ρ_{cald} (g*cm^{-3})	1.136
abs coeff. (mm^{-1})	0.090
F(000)	1576
θ range (deg)	2.28 – 25.05
no. of collected/unique rflns.	34649 / 7502
R_{int}	0.0365
data/restraints/params.	7502 / 61 / 507
goodness of fit on F^2	1.073
final R indices (I> 2σ(I))	R_1 = 0.0444, wR_2 = 0.1112
R indices (all data)	R_1 = 0.0580, wR_2 = 0.1198
largest diff. peak/hole (e/Å^3)	0.391 / -0.273

NMR Spectra

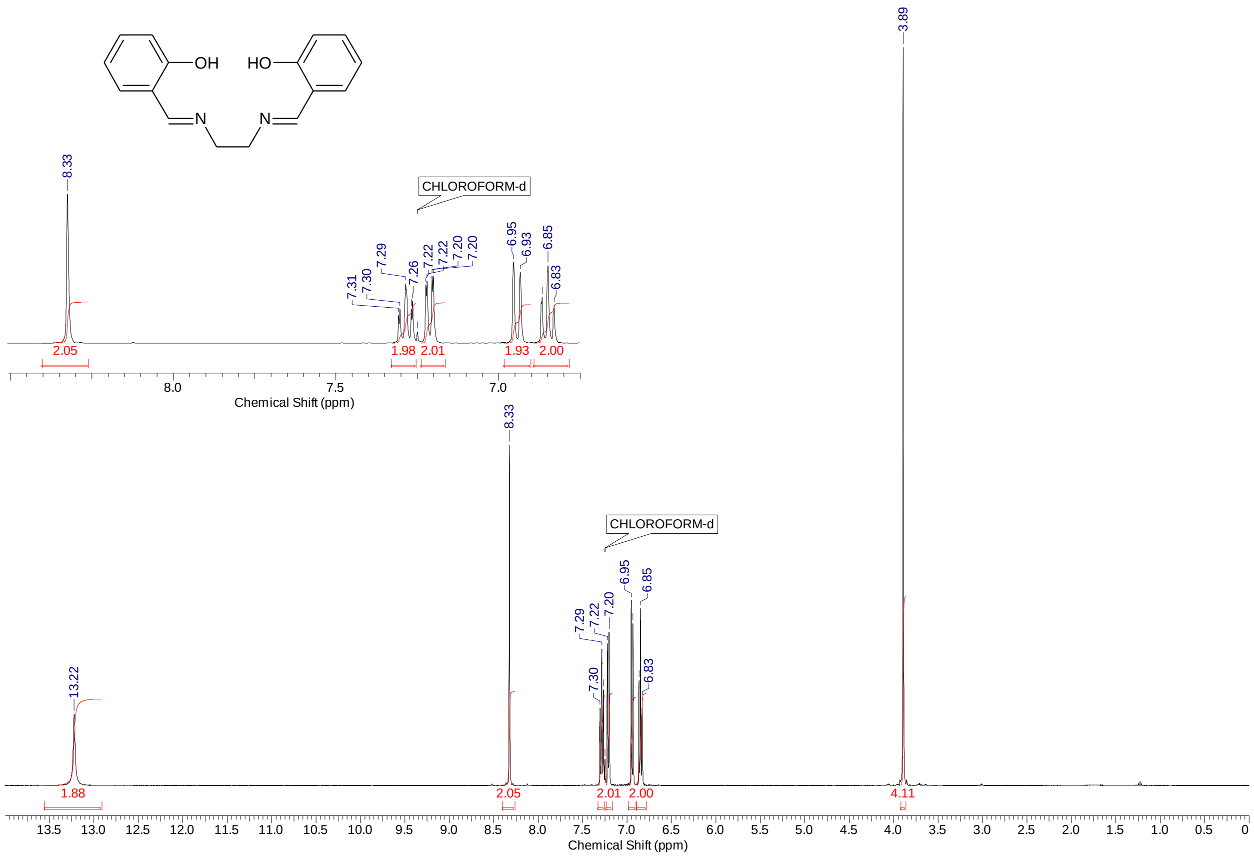

Figure S1. ^{1}H NMR spectrum of **1a** ($CDCl_3$, RT).

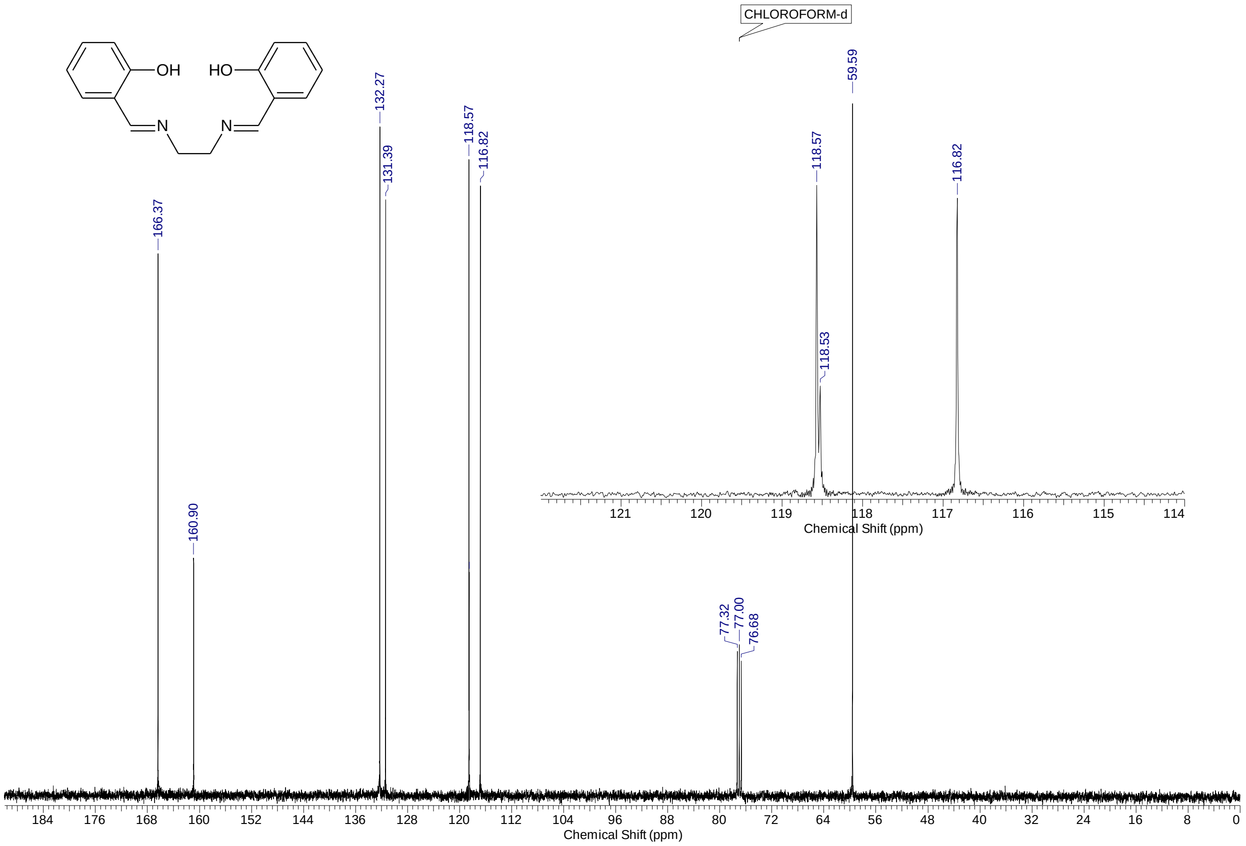

Figure S2. ^{13}C NMR spectrum of **1a** ($CDCl_3$, RT).

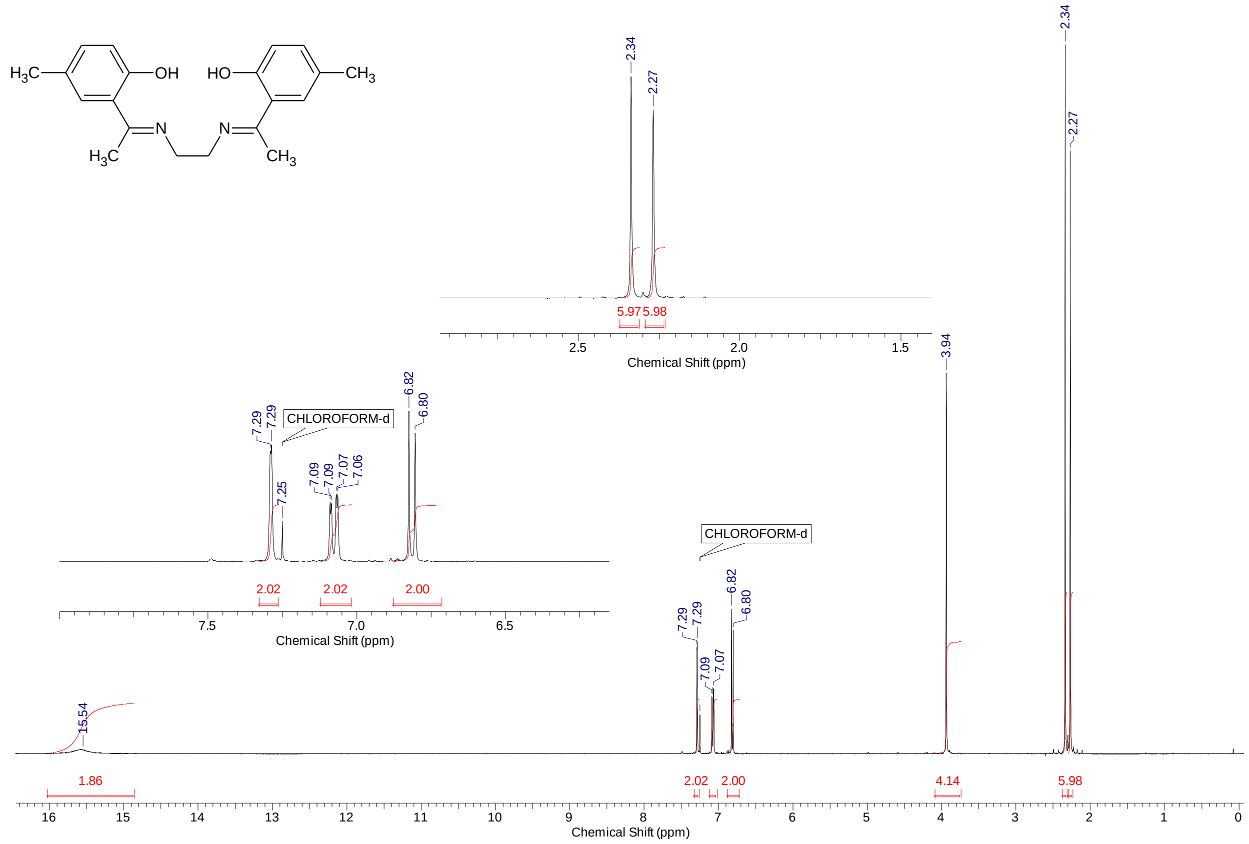

Figure S3. ^{1}H NMR spectrum of **1b** ($CDCl_3$, RT).

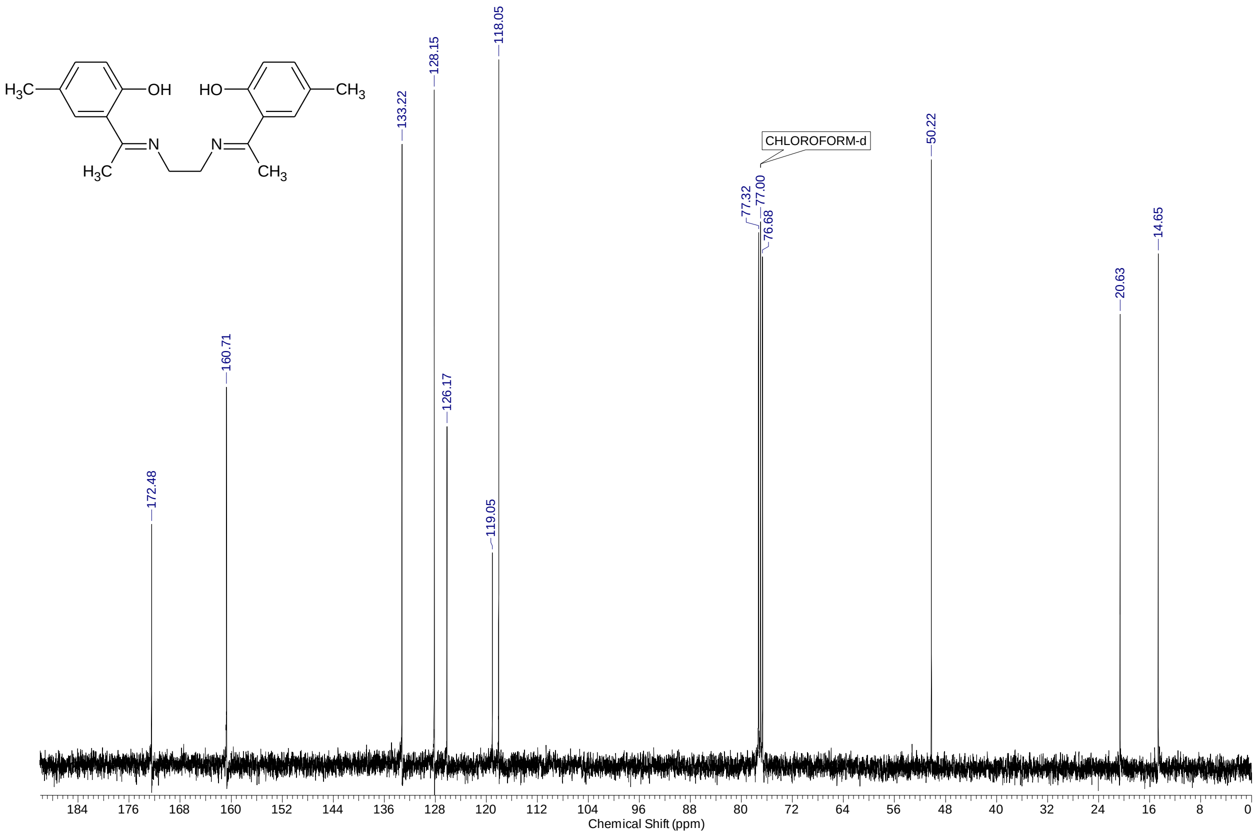

Figure S4. ^{13}C NMR spectrum of **1b** ($CDCl_3$, RT).

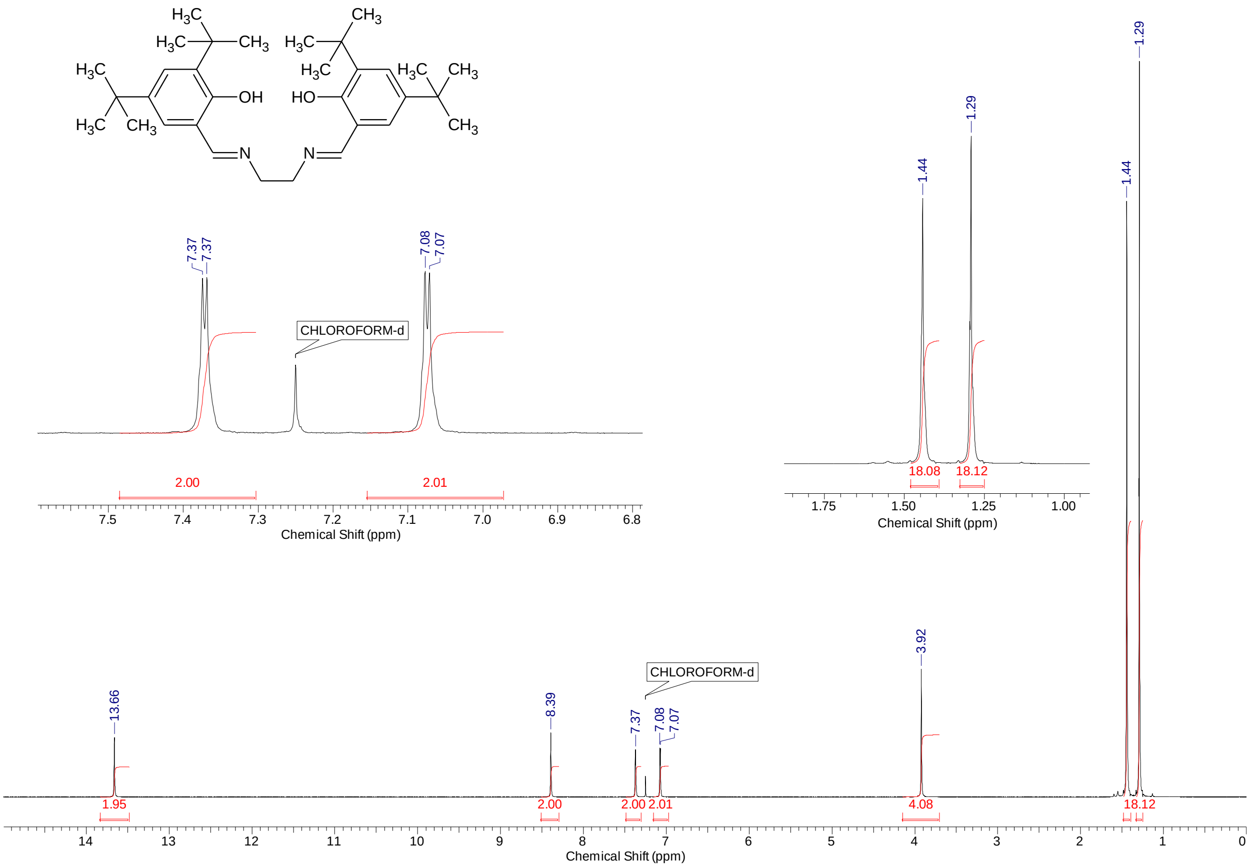

Figure S5. ^{1}H NMR spectrum of **1c** ($CDCl_3$, RT).

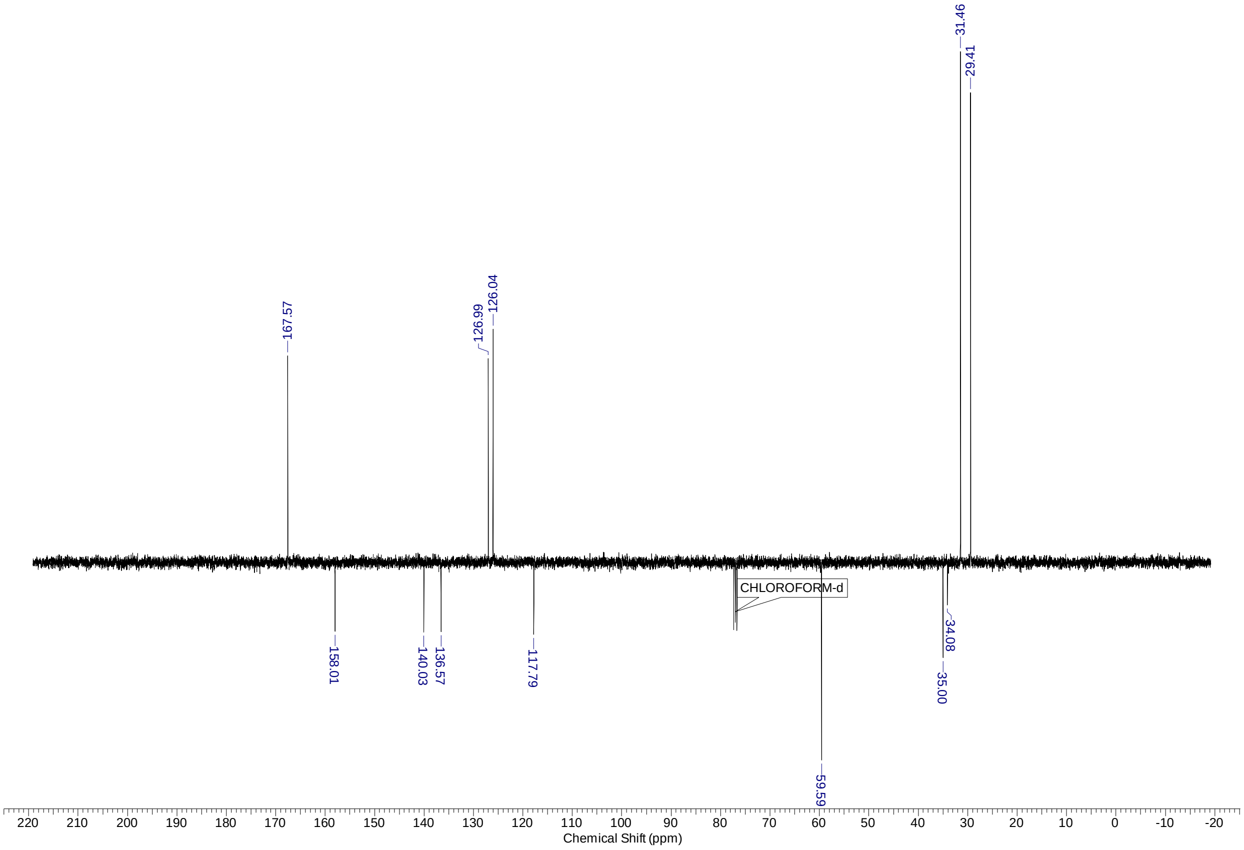

Figure S6. ^{13}C APT NMR spectrum of **1c** ($CDCl_3$, RT).

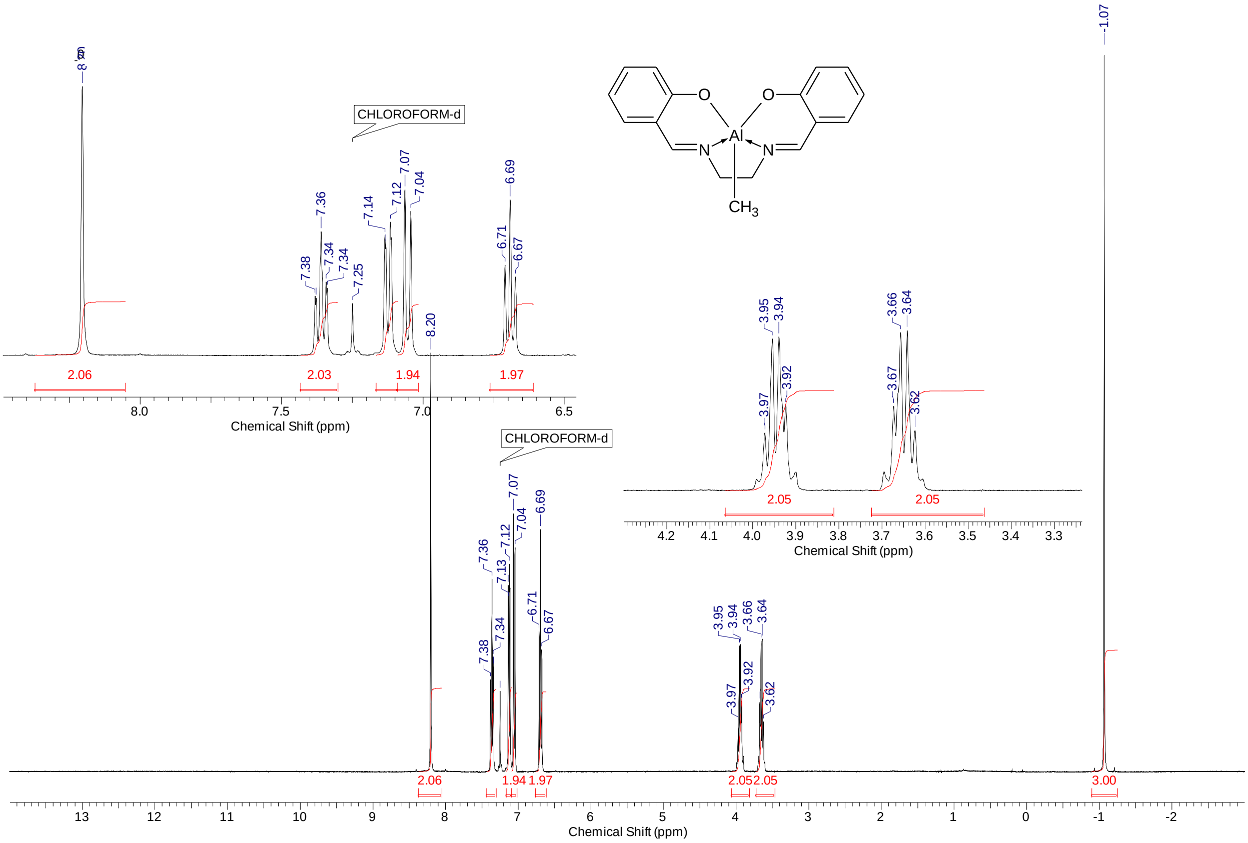

Figure S7. ^{1}H NMR spectrum of **2a** ($CDCl_3$, RT).

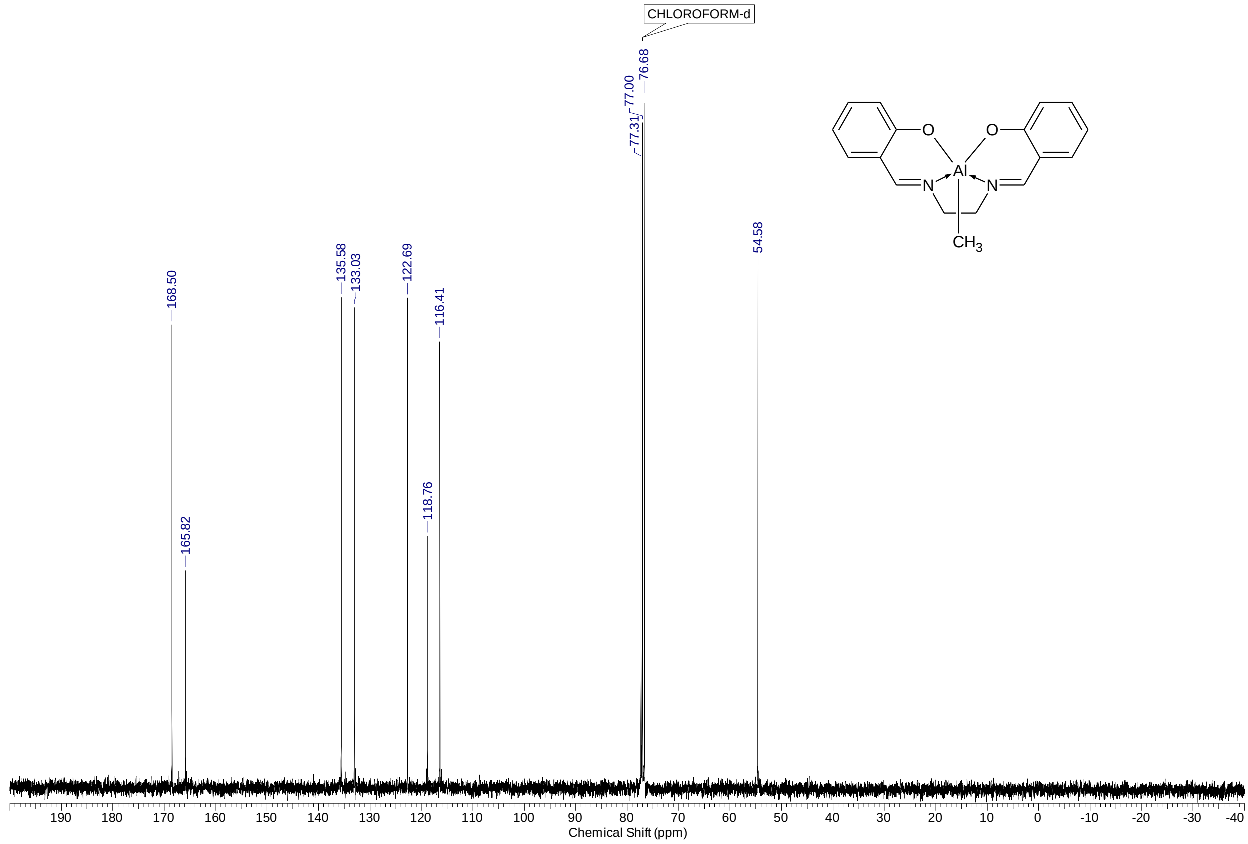

Figure S8. ^{13}C NMR spectrum of **2a** ($CDCl_3$, RT).

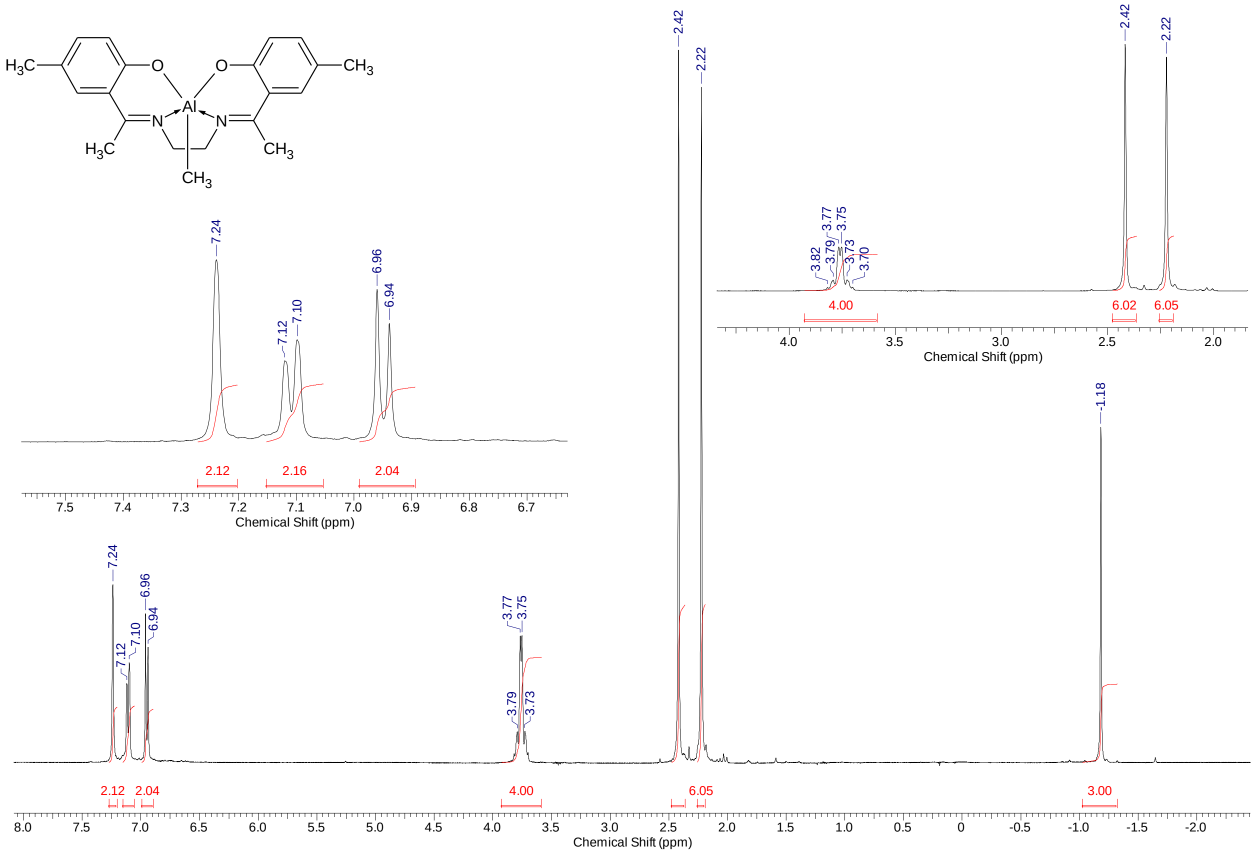

Figure S9. ^{1}H NMR spectrum of **2b** ($CDCl_3$, RT).

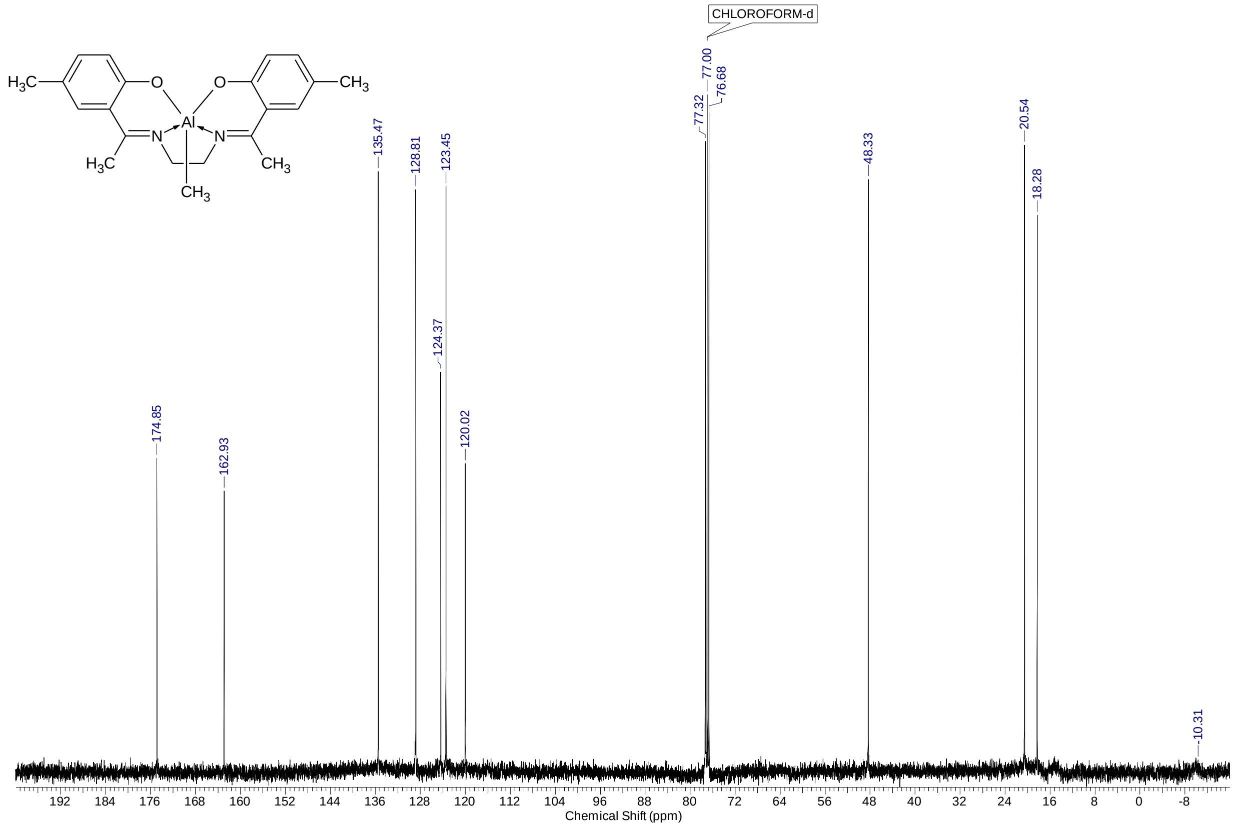

Figure S10. ^{13}C NMR spectrum of **2b** ($CDCl_3$, RT).

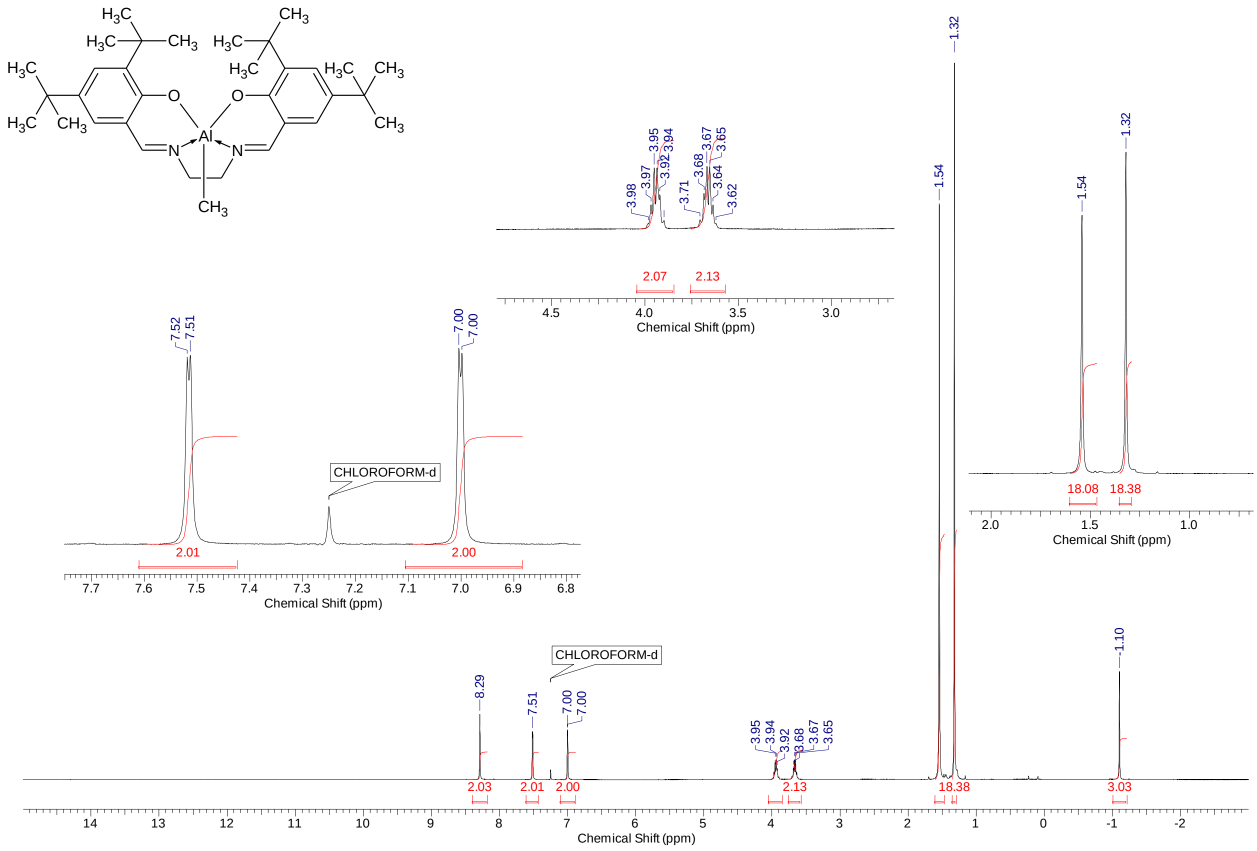

Figure S11. ^{1}H NMR spectrum of **2c** ($CDCl_3$, RT).

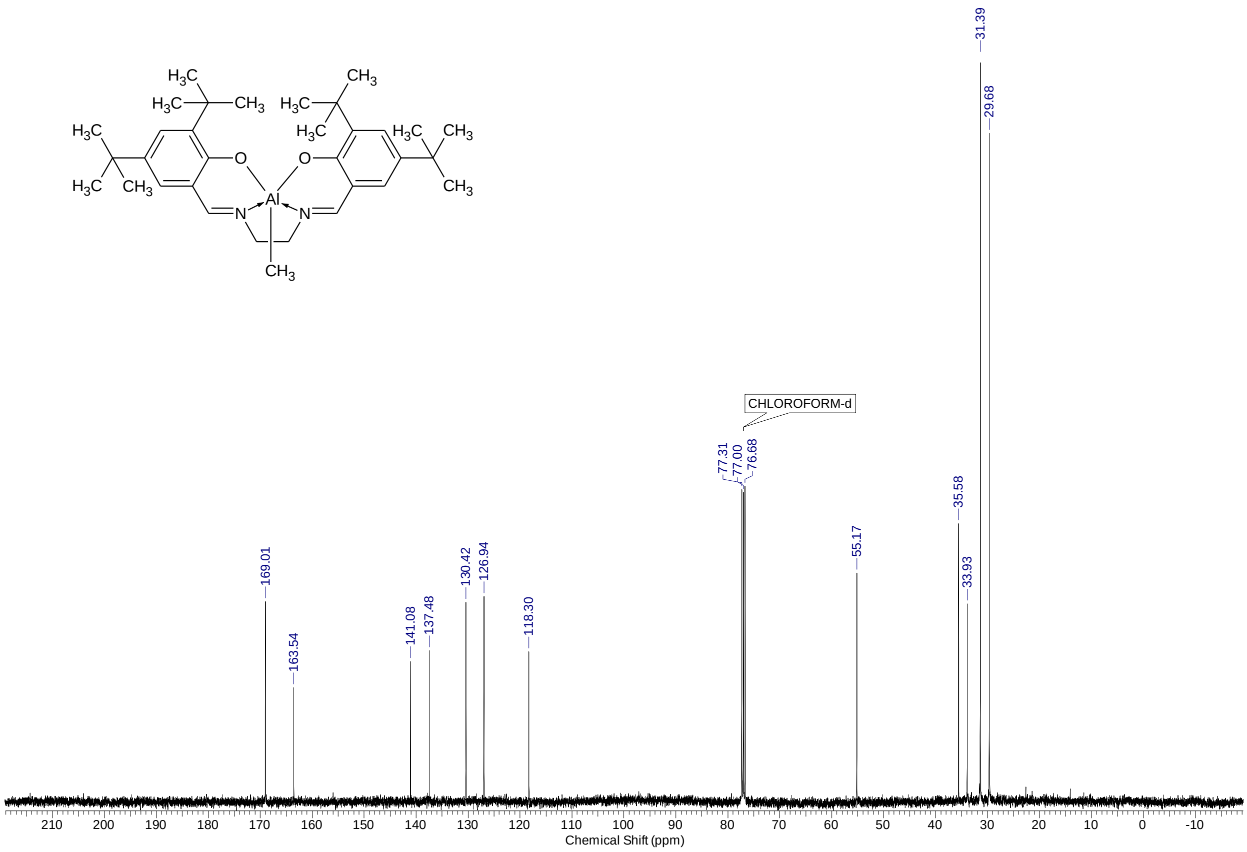

Figure S12. ^{13}C NMR spectrum of **2c** ($CDCl_3$, RT).

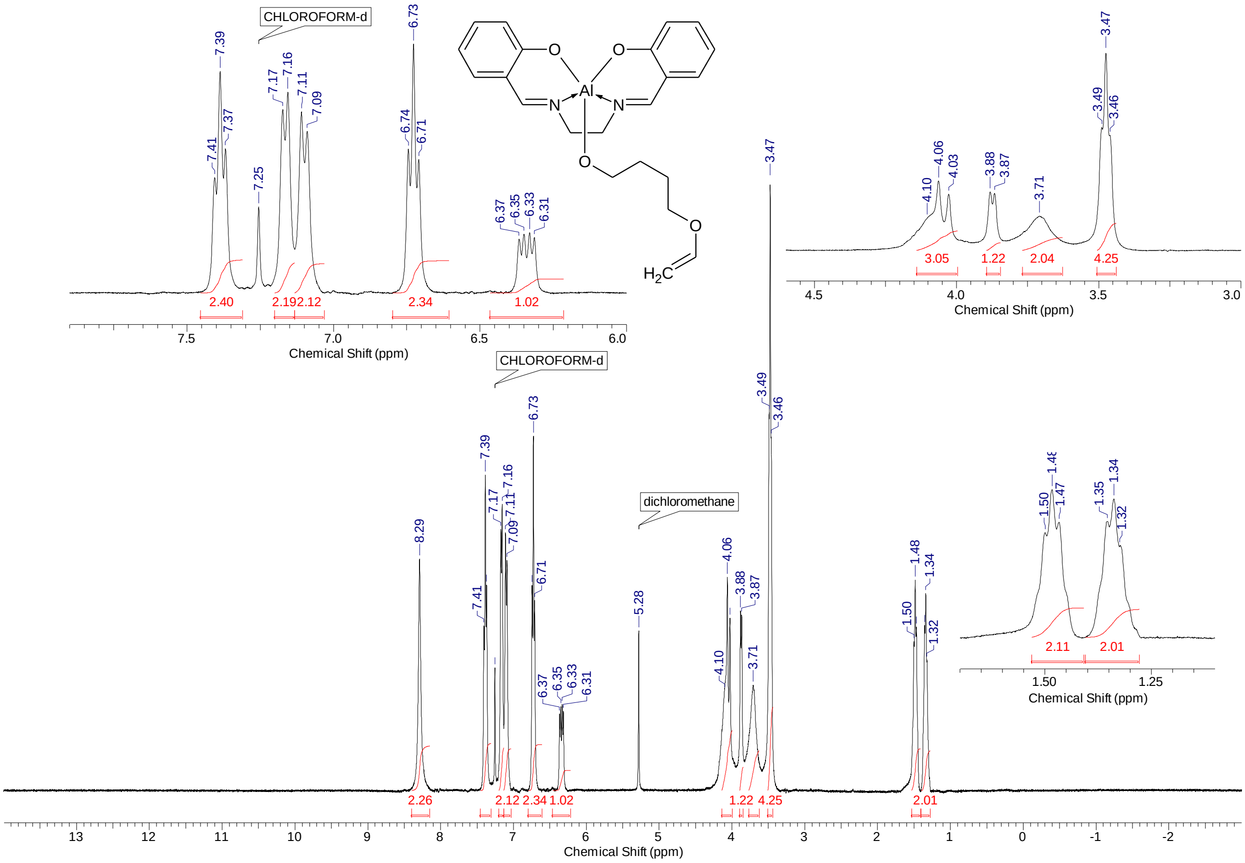

Figure S13. ^{1}H NMR spectrum of **3a** ($CDCl_3$, RT).

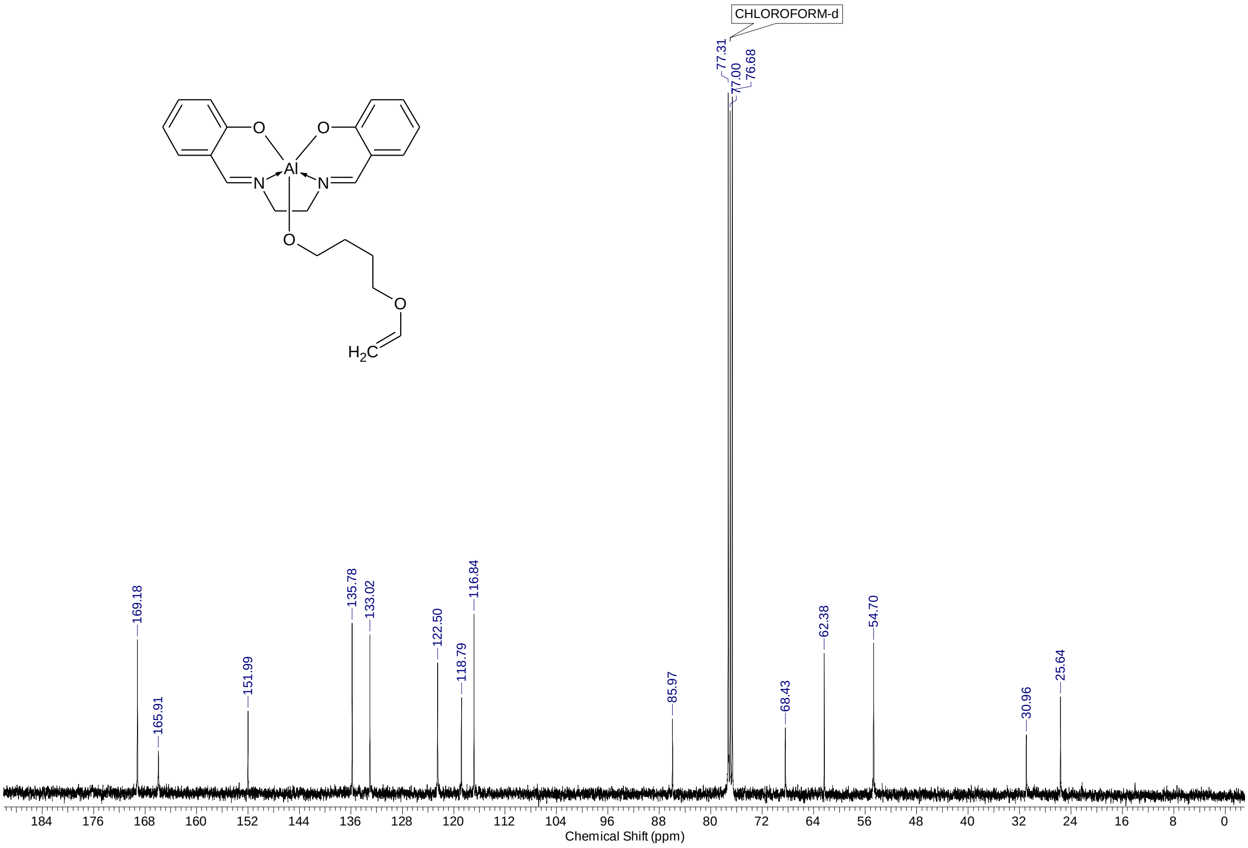

Figure S14. ^{13}C NMR spectrum of **3a** ($CDCl_3$, RT).

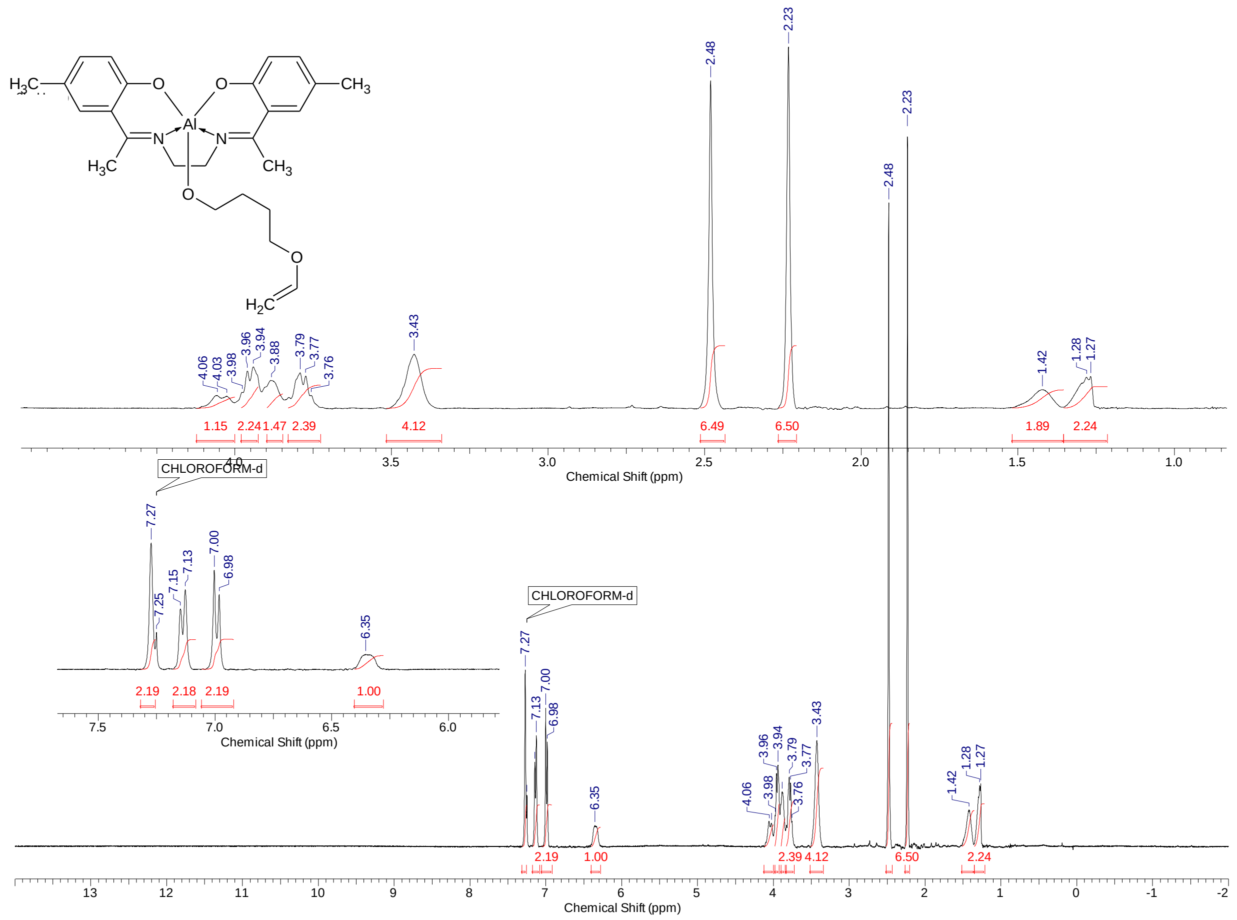

Figure S15. ^{1}H NMR spectrum of **3b** ($CDCl_3$, RT).

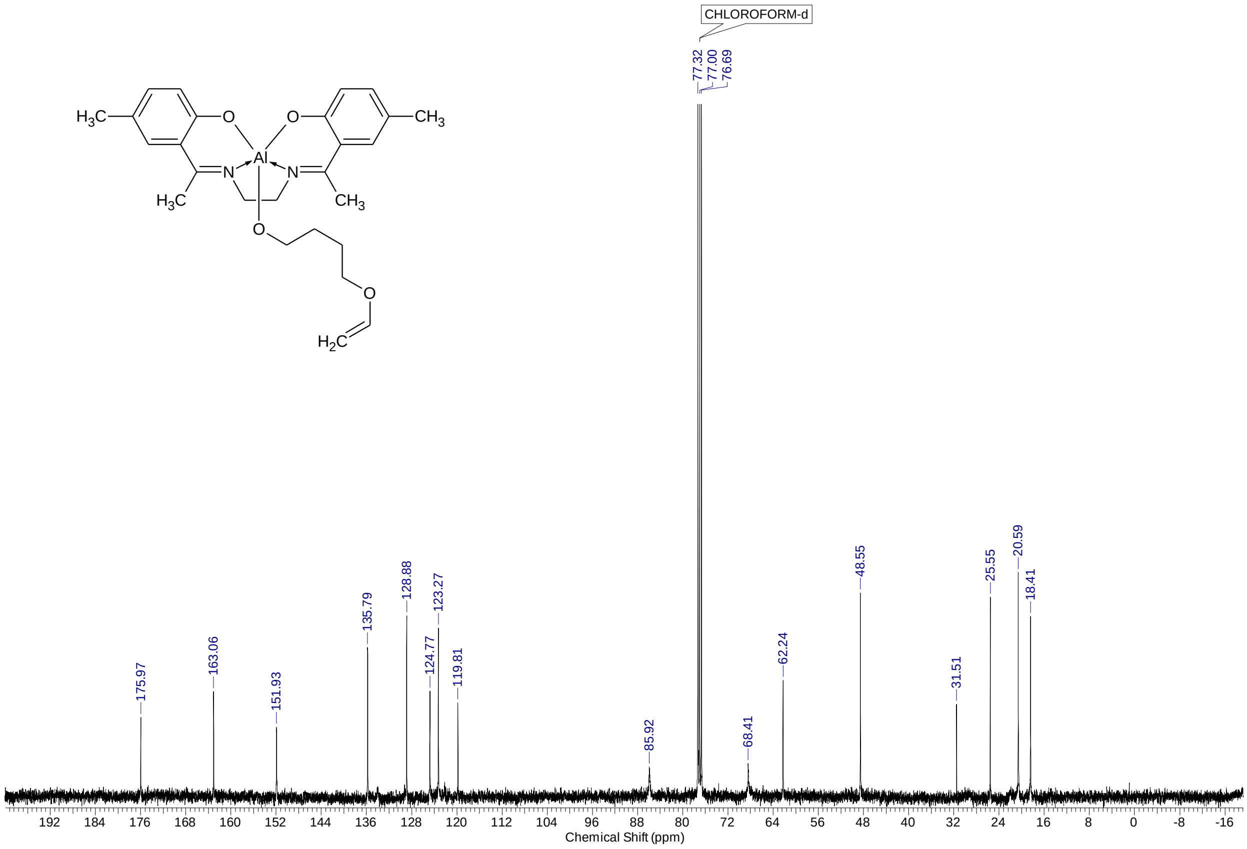

Figure S16. ^{13}C NMR spectrum of **3b** ($CDCl_3$, RT).

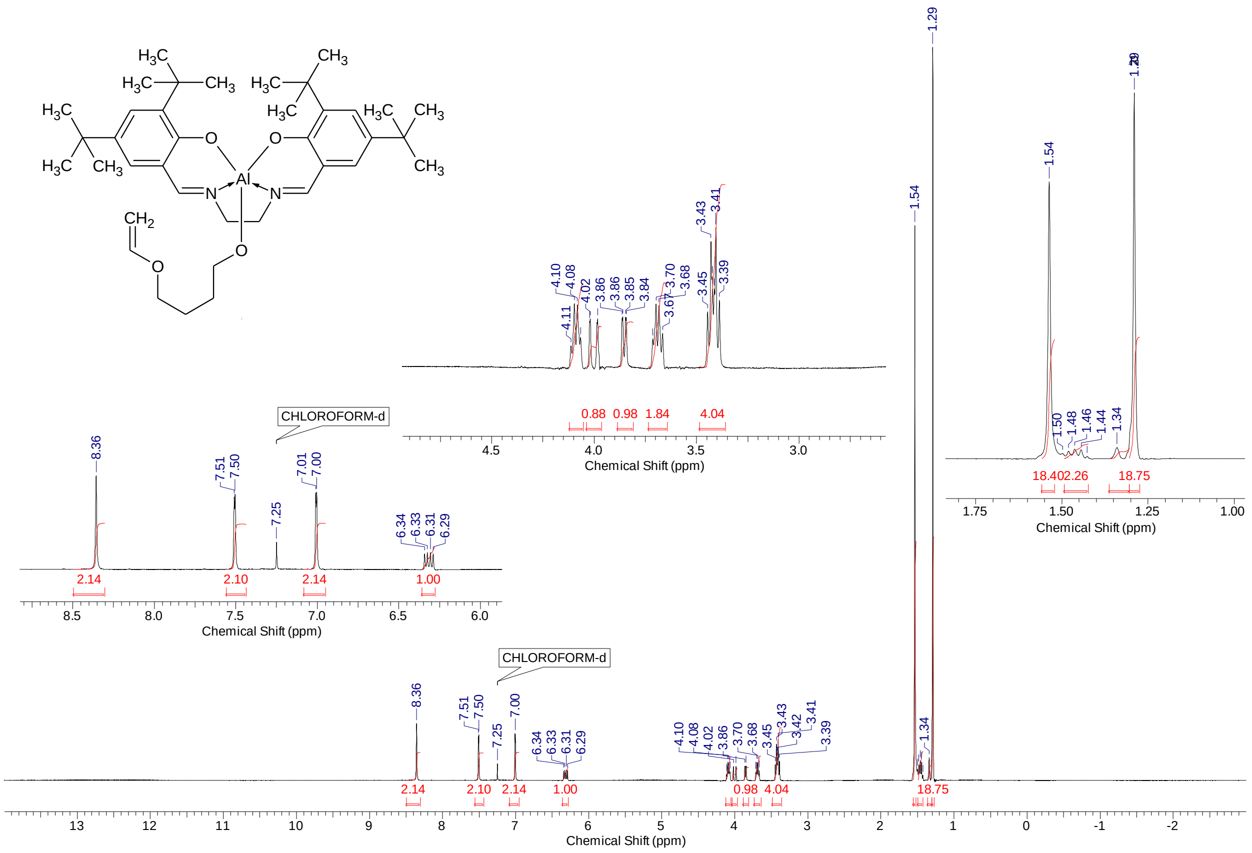

Figure S17. ^{1}H NMR spectrum of **3c** ($CDCl_3$, RT).

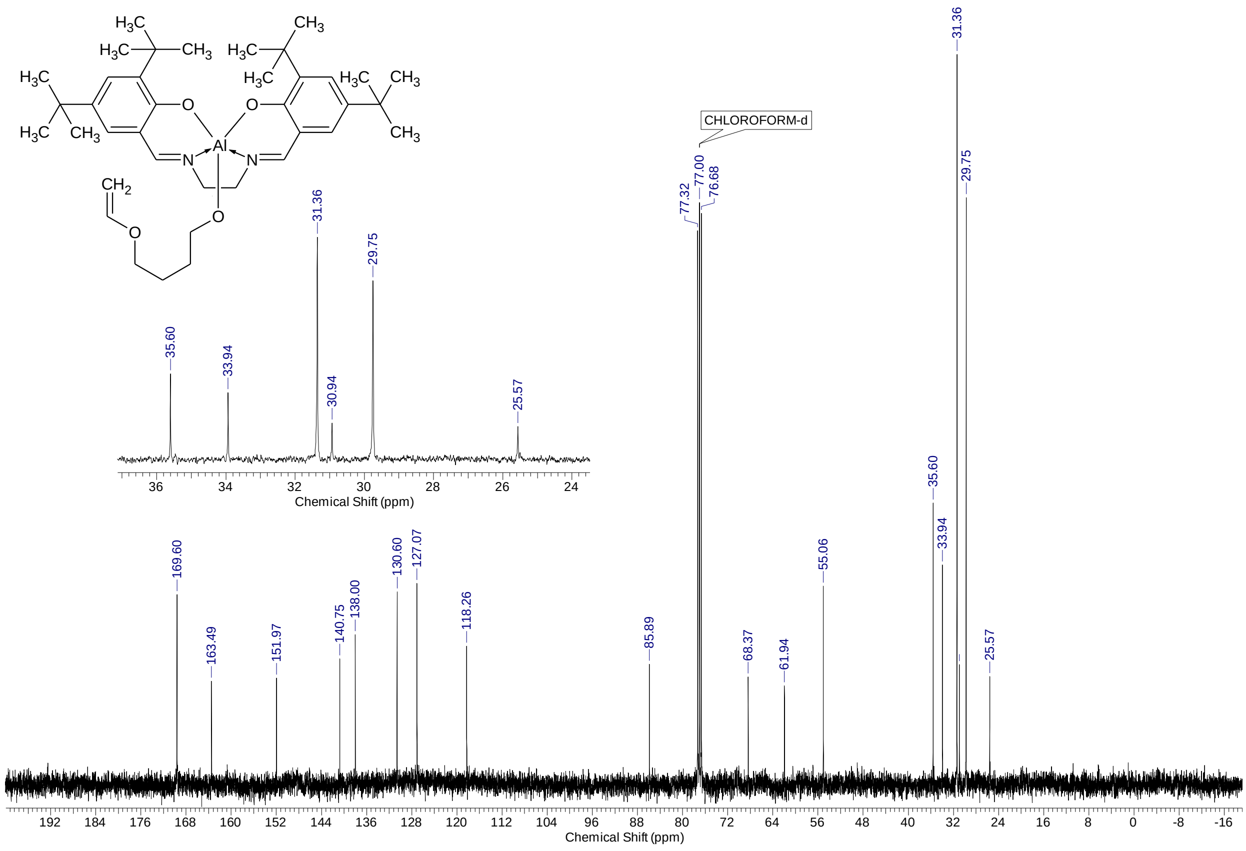

Figure S18. ^{13}C NMR spectrum of **3c** ($CDCl_3$, RT).

Polymerization data

Table S2. Bulk polymerization of *D,L*-lactide at different monomer/catalyst ratios for **3b** and **3c** at 130 ºC

Catalyst	[M]/[cat]	Time (h)	Conversion (%)	M_n (theor)[a] (g mol^{-1})	M_n[b] (g mol^{-1})	Đ	F_n[c] (%)
**3b**	100	0.4	99	14250	12480	1.92	75
	300	2	99	42800	34680	1.78	74
**3c**	100	17	97	14000	12120	1.79	79

[a] M_n(theor) = ([*D,L*-lactide]/[catalyst]) ×144×Conversion +115

[b] Experimental molecular weight determined by SEC versus polystyrene standards and corrected by a factor 0.58

[c] Calculated from ^{1}H NMR spectra as follows: $F_n = I(h)/I(a) \times 100$, see Figure S19 for details

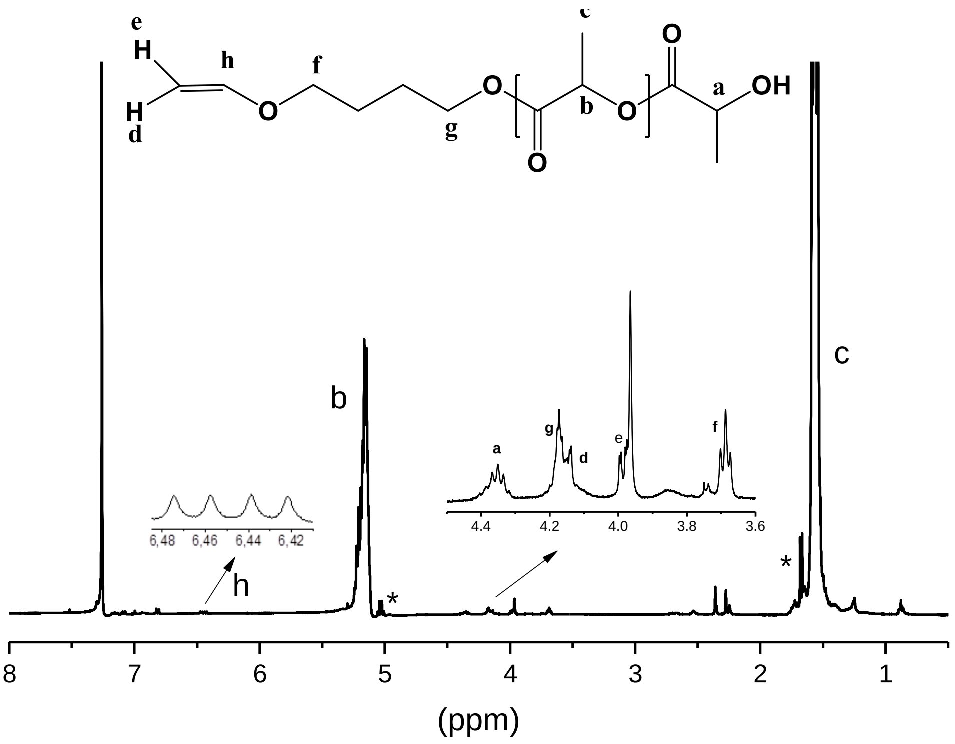

Figure S19. ^{1}H NMR spectrum of poly(*D*,*L*-lactide) synthesized with **3b** at 130 °C and at [*D*,*L*-lactide]/[catalyst] = 100.
